# Exploration of Immune-Modulatory Effects of Amivantamab in Combination with Pembrolizumab in Lung and Head and Neck Squamous Cell Carcinoma

**DOI:** 10.1158/2767-9764.CRC-24-0107

**Published:** 2024-07-17

**Authors:** Sun M. Lim, Seong-san Kang, Dong K. Kim, Soo-Hwan Lee, Chun-Bong Synn, Sujeong Baek, Seung M. Yang, Yu J. Han, Mi H. Kim, Heekyung Han, Kwangmin Na, Young T. Kim, Mi R. Yun, Jae H. Kim, Youngseon Byeon, Young S. Kim, Jii B. Lee, Min H. Hong, Joshua C. Curtin, Bharvin Patel, Isabelle Bergiers, Kyoung-Ho Pyo, Byoung C. Cho

**Affiliations:** 1 Division of Medical Oncology, Department of Internal Medicine, Yonsei Cancer Center, Yonsei University College of Medicine, Seoul, South Korea.; 2 JEUK Institute for Cancer Research, JEUK Co., Ltd., Gumi-si, South Korea.; 3 Severance Biomedical Science Institute, Yonsei University College of Medicine, Seoul, South Korea.; 4 Brain Korea 21 PLUS Project for Medical Science, College of Medicine, Yonsei University, Seoul, South Korea.; 5 Department of Research Support, Yonsei Biomedical Research Institute, Yonsei University College of Medicine, Seoul, South Korea.; 6 Yonsei New Il Han Institute for Integrative Lung Cancer Research, Seoul, South Korea.; 7 Janssen R&D, Spring House, Pennsylvania.; 8 Janssen R&D, Beerse, Belgium.

## Abstract

Immune checkpoint inhibitors are effective first-line therapy for solid cancers. However, low response rate and acquired resistance over time has led to the need for additional therapeutic options. Here, we evaluated synergistic antitumor efficacy of EGFR × MET targeting bispecific antibody, amivantamab with PD-L1 immunotherapy, pembrolizumab in head and neck squamous cell carcinoma (HNSCC) and lung squamous cell carcinoma tumor–bearing humanized patient-derived xenograft (PDX) models. We demonstrated that pembrolizumab or amivantamab alone was ineffective and that combination treatment induced a significant reduction of tumor growth in both models (*P* < 0.0001 and *P* < 0.01, respectively). It appeared that combination of amivantamab and pembrolizumab significantly enhanced infiltration of granzyme B–producing CD8 T cells was in the TME of HNSCC PDX (*P* < 0.01) and enhanced neoantigen-associated central memory CD8 T cells in circulating immune cells. Analysis of single-cell RNA transcriptomics suggested that the tumor cells dramatically upregulated EGFR and MET in response to PD-L1 immunotherapy, potentially creating a metabolic state fit for tumor persistence in the tumor microenvironment (TME) and rendered pembrolizumab ineffective. We demonstrated that EGFR^HIGH^MET^HIGH^ subcluster displayed an increased expression of genes implicated in production of lactate [SLC16A3 and lactate dehydrogenase A (LDHA)] compared to the EGFR^LOW^MET^LOW^ cluster. Accumulation of lactate in the TME has been associated with immunosuppression by hindering the infiltration of tumor killing CD8 T and NK cells. This study proved that amivantamab reduced glycolytic markers in the EGFR^HIGH^MET^HIGH^ subcluster including SLC16A3 and LDHA and highlighted remodeling of the TME by combination treatment, providing rationale for additional therapy of amivantamab with PD-1 immunotherapy.

**Significance:**

Amivantamab in synergy with pembrolizumab effectively eradicated EGFR^HIGH^MET^HIGH^ tumor subcluster in the tumor microenvironment of head and neck squamous cell carcinoma and overcame resistance against anti-PD-1 immunotherapy.

## Introduction

The development of immune checkpoint inhibitors (ICIs) has revolutionized the treatment of multiple solid cancer types, and ICIs have emerged as an effective treatment option even in the first-line setting (1). Pembrolizumab alone or in combination with cytotoxic chemotherapy has been approved as an effective first-line therapy in both non–small cell lung cancer (NSCLC) and head and neck squamous cell carcinoma (HNSCC). However, the benefit of pembrolizumab is often diminished by the low response rate and a limited proportion of responders, and this has given rise to the need for developing different combination treatment regimens to improve antitumor immune response (2). Understanding the nonresponse to anti-programmed cell death protein 1 (PD-1) or anti-programmed death-ligand 1 (PD-L1) treatment is multifaceted (3), and low PD-L1 expression (4), low mutational burden (5), and deficiencies in antigen presentation (6) were previously studied. However, the tumor microenvironment consists of various immune cells which delicately interact with each other to potentiate anticancer immune responses (7). In order for anti-PD-1/L1 treatment to work, tumor-reactive T cells need to be present in the tumor, but their presence is not sufficient. Effector CD8^+^ T cell responses are regulated also by innate immune cells such as natural killer (NK) cells, dendritic cells (DCs) and macrophages (8). Therefore, augmenting the innate immune cell functions to boost T-cell checkpoint inhibitor response may provide further therapeutic benefits.

Unlike lung adenocarcinoma, lung squamous cell carcinoma is not known to be druggable with actionable genomic alterations. The activity of a single agent pembrolizumab is only modest, and there is a huge clinical unmet need for developing new therapeutic strategies. HNSCC is known to have only one targeted therapy approved, which is cetuximab, an epidermal growth factor receptor (EGFR) monoclonal antibody. EGFR and hepatocyte growth factor receptor (MET) overexpression is commonly noted in approximately 90% cases of HNSCC (9), which could be targeted by amivantamab. Likewise, the activity of pembrolizumab alone is limited in HNSCC. Thus, we chose to investigate the synergistic effects of the combination treatment and provide rationale for treatment regimen in these two tumor types.

Amivantamab is a fully human bispecific antibody that binds to the EGFR and MET receptor to inhibit ligand binding, promote downregulation of cell surface receptors, and induce Fc-dependent immune activity (10–13). Amivantamab displays a tolerable safety profile, enabling combinatorial approaches, and has shown antitumor activity in diverse EGFR- and MET-driven NSCLC (14, 15). Amivantamab is approved for the treatment of patients with locally advanced or metastatic NSCLC with EGFR exon 20 insertion mutations, whose disease progressed on or after platinum-based chemotherapy (14, 16–18). By inhibiting EGFR and c-Met signaling functions, either by blocking ligand-induced activation or inducing receptor degradation, amivantamab may disrupt these signaling pathways and prevent tumor growth and progression. Furthermore, interesting features of amivantamab are the Fc-dependent effector mechanisms including antibody-dependent cellular cytotoxicity (ADCC) and antibody-dependent cellular phagocytosis (ADCP). Amivantamab has shown to increase immune-mediated antitumor activity via ADCC, ADCP, and trogocytosis.

In this study, we hypothesized that combination of amivantamab with pembrolizumab would enhance antitumor efficacy. Therefore, we aimed to investigate the comprehensive immune-modulatory effects of the combination regimen in our established humanized mice models of HNSCC and lung squamous cell carcinoma (LUSC) harboring EGFR and/or MET expression.

## Materials and Methods

### Patient-derived xenograft selection

Internal patient-derived xenograft (PDX) library tumors were screened for EGFR and MET expression by immunohistochemistry (IHC; Supplementary Fig. S1A and S1B). Immunohistochemical staining of EGFR and MET were quantified using intensity scores and compared between the models prior to selecting the tumors with dual expression of EGFR and MET among LUSC and HNSCC. By intensity score, HNSCC model (YHIM-3003) showed co-expression of EGFR (average score of 157.8 ± 21.3) and MET (average score of 21.4 ± 12.6; Supplementary Fig. S1C). LUSC model (YHIM-2010) showed both expression of EGFR (average score of 199.4 ± 48.82) and MET (average score of 161.4 ± 34.1). LUSC model was previously deemed insensitive to treatment of pembrolizumab (Supplementary Fig. S1D and S1E). YHIM-3003 and YHIM-2010 did not exhibit EGFR mutations (Supplementary Fig. S1D). Additionally, YHIM-3003 and YHIM-2010 demonstrated the most consistent and stable tumor development compared to the other models.

### PDX inoculation and establishment of humanized mice

All patient samples were obtained from patients at Yonsei University Severance Hospital (Seoul, Republic of South Korea). The study protocol has been approved by the Institutional Review Board of Severance Hospital (IRB no. 4-2016-0788) and all patients have provided written informed consent. This study conforms to the principles set out in the World Medical Association Declaration of Helsinki and the U.S. Department of Health and Human Services Belmont Report. Tumors from patients with HNSCC (YHIM-3003) and LUSC (YHIM-2010) were engrafted into 6- to 8-week-old female humanized CD34^+^ NOD-scid IL2Rgamma^null^ (Hu-CD34-NSG, purchased from The Jackson Laboratory, Maine, US) mice to generate humanized HNSCC and LUSC PDX mice models. Animal procedures were approved by the Institutional Animal Care and Use Committee (IACUC) and Animal Research Committee at Yonsei University College of Medicine (Seoul). After removal of the necrotic and supporting tissues from core biopsy specimens, small specimens of the tumor tissue (3 mm × 3 mm × 3 mm) from each patient were implanted subcutaneously in one to two mice. The tumor was excised after reaching 1.5 cm in diameter and reimplanted into humanized mice in small specimens (3 mm × 3 mm × 3 mm). The animals were randomly grouped based on tumor volume and 15 mice were allocated per group. Five mice in each group were designated as internal sacrifice group at day 5. Tumor grafts were measured once the tumor became visibly stable and palpable.

### *In vivo* drug treatment and preparation

Drug treatment initiated when the tumor reached 200 mm^3^ for both models. The animals were treated with multiple doses of drugs until the endpoint of the *in vivo* assay as the following. Amivantamab was administered at 10 and 30 mg/kg (mpk) biweekly (BIW(Backspace) via intraperitoneal injection (i.p.) in combination with pembrolizumab (10 mpk, i.p.) given every 5 days (Q5D)) for the LUSC PDX (YHIM-2010). For the duration of HNSCC PDX (YHIM-3003) experiment, 10 mpk of amivantamab was administered in combination with pembrolizumab. The treatment groups consisted of vehicle, amivantamab, pembrolizumab, and combination. Each group consisted of 15 mice where five mice were ethically sacrificed at day 5 and the remaining 10 mice persisted with treatment until the end of survival assay. Amivantamab and pembrolizumab were prepared by diluting with antiseptic grade of Hanks’ Balanced Sat Solution (HBSS, Life Technologies, NY, USA) for intraperitoneal injection. The drug solution was prepared on the day and mixed vigorously before injection. Amivantamab was provided by Janssen Corporation.

### Sample preparation

Five mice from each group were ethically sacrificed for collection of blood, tumor, and spleen samples at day 5 of the *in vivo* experiments. The tumor samples were excised into portions. A proportion of tumor tissue was fixed in 10% formalin and made into formalin-fixed, paraffin-embedded (FFPE) blocks to generate slides for multiple IHC. The remaining proportion was divided to be used for single-cell RNA sequencing and flow cytometry. Spleen samples were processed for FFPE blocks and flow cytometry. For flow cytometry analysis, the tumor was chopped into small pieces using sterile surgical blades in PBS and enzymatically dissociated into single cells with collagenase (Worthington Biochemical, New Jersey, US) at 37°C for 1 hour and filtered through 70 μm cell strainer. Spleen samples were dissociated by gently tapping with syringe stopper in PBS before filtering through 70 μm cell strainer. Dissociated samples were frozen in FBS + 10% DMSO stored at −80°C until required for analysis. These samples were processed for TME, flow cytometry, and single-cell RNA sequencing analysis.

### Multiplex immunohistochemical staining

For multiplexed immunohistochemical staining, BOND RX Fully Automated Research Stainer (21.2821, Leica Biosystems, Nubloch, Germany) and Opal Polaris 7 Color IHC Detection Kit (P-000003, Akoya Biosciences, Massachusetts, US) were utilized. All procedures were performed according to the manufacturer’s instructions. In brief, deparaffinized sections were incubated with citrate- or Tris-based antigen unmasking solutions (for heat-induced epitope retrieval) at 98°C for 20 minutes. Sections were then treated with hydrogen peroxide and a protein-blocking reagent to prevent the nonspecific binding of antibodies to the sections. Sections were sequentially treated with the primary antibodies, horseradish peroxidase (HRP)-conjugated antibodies, and specific fluorophores to detect target biomarkers. Multiple staining rounds were performed using the following antibodies: anti-CD68 (76437, Cell Signaling Technology, Massachusetts, US), anti-PD-L1 (ACI 3171A, Cell Signaling Technology), anti-granzyme B (Cell Signaling Technology), anti-CD16 (24326, Cell Signaling Technology), anti-CD163 (NCL-L-CD163, Leica Biosystems), anti-CD11c (45581, Cell Signaling Technology), anti-CD4 (ab181724, Abcam, Cambridge, United Kingdom), anti-CD8 (CD8-4B11-L-CE, Leica Biosystems), anti-PD-1 (ab137132, Abcam) anti-FoxP3 (98377; Cell Signaling Technology), anti-PanCK (AE1/AE3-601-L-CE; Leica Biosystems). Tissue sections were counterstained with Spectral 4′,6-diamidino-2-phenylindole (FP1490; Akoya Biosciences).

### Imaging analysis

Images of the whole tissue contents were generated using whole-slide scanning by using Vectra Polaris Automated Quantitative Pathology Imaging System (CLS143455, Akoya Biosciences). Multispectral images for analysis (up to 98 region of interest sites, Supplementary Data) were defined and selected within the whole tissue using Phenochart whole slide contextual viewer software (version 1.12, Akoya Biosciences). The inForm software (version 2.6, Akoya Biosciences), equipped with an integrated algorithm for tissue analysis, was employed to transform the multispectral image data into numerical data. These data encompassed both numerical and spatial information regarding the tumor nest and stromal region, defining the cell components (nuclear, cytosolic, and membrane margins), classifying the cell populations and the intensities of each marker. Cell populations were discerned based on the distinctive patterns of CD marker expression, each exhibiting unique cellular properties, such as nuclear, cytosolic, and membranal sizes and shapes (CD4/8 T cells, CD68 for macrophages, Pan-CK for epithelial cells or cancer cells and FoxP3 for regulatory T cells). The differentiation between the tumor nest and stroma, as well as the area calculations, were done based on the pan-cytokeratin staining patterns by utilizing an algorithm integrated into the inForm software. The integrated matrix files were derived from the segmentation of cells and tissues from the tissue microarray data. These data included the fluorescence intensity in the nucleus and cytosol. The extracted CSV file was converted into an FCS file using the R program. The FCS file was subsequently converted into a raw file for cytometric image analysis using FlowJo (version 10.9.0).

### Flow cytometry analysis

Tumor cells and splenocytes were collected at day 5 of *in vivo* and analyzed using flow cytometry. For immune assay, dissociated single cells washed with fluorescence-associated cell sorting (FACS) buffer (PBS containing 1% BSA, 0.01% sodium azide, 0.5 mmol/L EDTA) and blocked with FcR Blocking Reagent (Miltenyi Biotec, North Rhine-Westphalia, Germany) at room temperature for 20 minutes prior to antibody staining for analysis of T cells and myeloid cells using panels shown in Supplementary Table S1A. Splenocytes were stained using the panel as described in Supplementary Table S1B. Fixation and permeabilization for intracellular staining were prepared with True-Nuclear Transcription Factor buffer at room temperature for 30 minutes. Antibodies and gating strategies for identification of immune cell are described in supplementary data. Multicolor flow cytometric analysis was performed using BD LSRfortessa X-20 (BD Bioscience, New Jersey, USA). FlowJo software (FlowJo LLC, Oregon, USA) was used for data acquiring and analysis.

### Cell lines

The LUSC cell lines, EBC-1 (RRID: CVCL_2891) and H1703 (CRL-5889), were purchased from American Type Culture Collection (ATCC, Manassas, VA, USA). The cells were cultured and maintained in HyClone RPMI-1640 (Cytiva, Massachusetts, US) supplemented with 10% fecal bovine serum (Cytiva) and 1% antibiotic/antimycotic solution (Cytiva) in a humidified incubator with 5% CO_2_.

### Western blot reagents

Amivantamab was kindly provided by Janssen Corporation for all *in vivo* and *in vitro* studies. Following primary antibodies were used for protein detection. Anti-actin (#A3854, 1:1,000) was purchased from Sigma Aldrich. Anti-EGFR (#2232, 1:1,000), anti-p-EGFR (#2234, 1:1,000), anti-LDHA (#3582, 1:1,000), anti-MET (#8198, 1:1,000) and anti-p-MET (#3077, 1:1,000) were purchased from Cell Signaling Technology. Anti-SLC16A3/MCT4 (ab74109, 1:1,000) was purchased from Abcam. Secondary HRP-conjugated anti-rabbit (#7074, 1:1,000) or anti-mouse IgG (#7076, 1:1,000) purchased from Cell Signaling Technology and ECL System (#1707062, Bio-Rad) were used for protein detection.

### Single-cell RNA library construction and sequencing

The tumor samples were dissociated using gentleMACS human tumor dissociation kit (Miltenyi Biotec) then processed prior to single-cell library preparation following the manufacturer’s guidelines (10X Genomics, California, US). Volumes for each sample were calculated for target capture of 10,000 cells. The processed samples were prepared for gene expression analysis using Chromium Single Cell 5′ Reagent Kits (10X Genomics). Single-cell library was then outsourced for single-cell RNA sequencing at Macrogen (Korea). Sequencing was performed to achieve read depth of more than 50,000. FASTQ files were then processed for mm10- and hg19-based mapping to distinguish mouse- and human-derived genome. Read counts and merging of the samples were executed via Cell Ranger (7.1.0). Scanpy was used for further quality control including filtering (minimum of 200 genes in at least three cells, with <20% of mitochondrial reads as cutoff) and normalization of cells (Harmony), batch correction, and clustering. Analysis of gene expression in HNSCC and LUSC models was visualized using heatmaps, violin, and dot plots via Seurat (version 4.9.9).

### Tumor-associated tetramer assay

Tetramer peptides were generated to compare the proportion of tumor-specific T lymphocytes between the treatment groups. CEA (HLA-A*24:02 TYACFVSNL) were outsourced from Biolegend (California, US). Preparation of Flex-T Tetramer is available on https://www.biolegend.com/en-us/protocols/flex-t-tetramer-preparation-and-flow-cytometry-staining-protocol.

### Statistical analysis

All statistical calculations were performed using Prism 9.0 (RRID:SCR_00279, GraphPad Software Inc, California, USA). Significant differences were evaluated using the unpaired samples *t* test and one-way analysis of variance. All experiments were performed at least in duplicates where possible, and error bars represent the mean ± SD unless indicated differently in the figure legends or graphs. Applied statistical tests and *P* value significances are described in each figure legend.

### Data availability

The data generated in this study are available upon request from the corresponding author.

## Results

### Amivantamab showed synergistic antitumor efficacy with pembrolizumab in head and neck squamous and lung squamous humanized mice models

**Figure 1:**
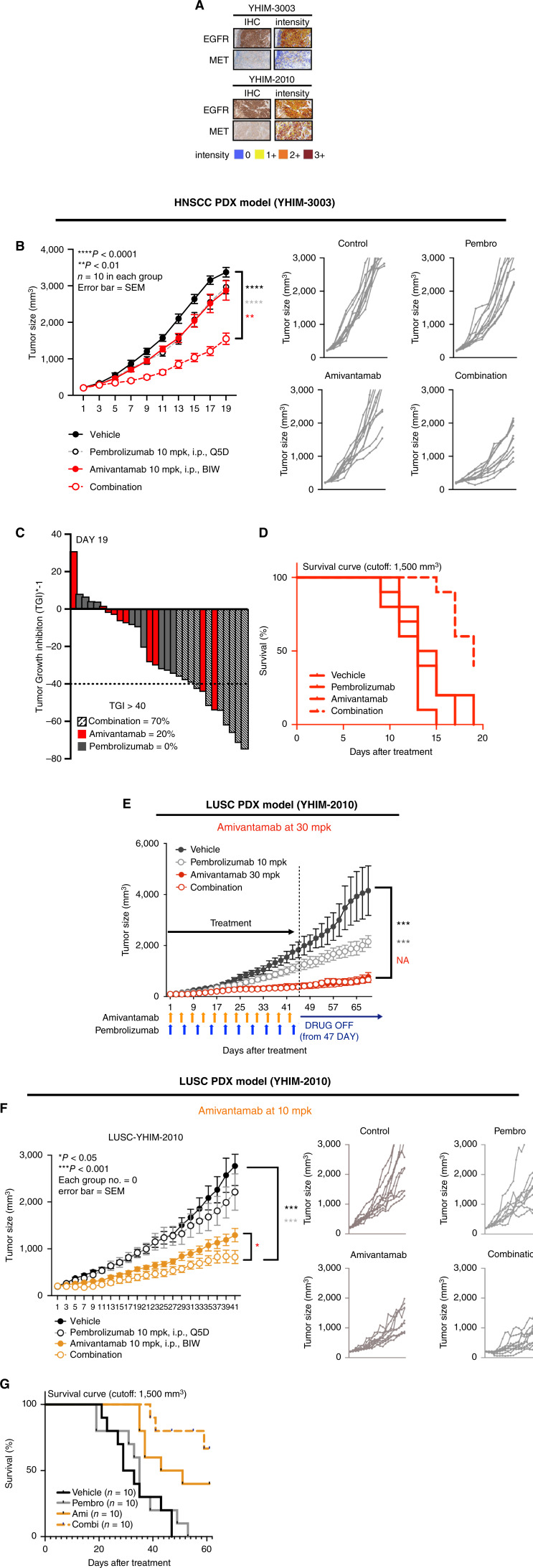
Antitumor effects of amivantamab w/wo pembrolizumab in HNSCC and LUSC tumor–bearing humanized PDX preclinical models. **A,** The intensity of EGFR and MET in YHIM-3003 (HNSCC) and YHIM-2010 (LUSC) tumors. **B,** The tumor progression of YHIM-3003 model over 19 days showing significant tumor regression by the combination treatment of amivantamab (30 mpk) and pembrolizumab (10 mpk) compared to single treatment of amivantamab and pembrolizumab (*P* < 0.001, *n* = 10 in each group). **C,** Tumor growth inhibition represented in a waterfall plot of HNSCC PDX model at day 19. **D,** Survival curve of HNSCC PDX demonstrating improved survival by the combination treatment (*n* = 10 in each group). **E,** Treatment of LUSC PDX using amivantamab at 30 mpk in combination with pembrolizumab (10 mpk, *n* = 10 in each group). No rebound of tumor growth was observed after the termination of amivantamab and combination treatment. **F,** Tumor regression in LUSC PDX showing significant tumor reduction by combination treatment of amivantamab (10 mpk) and pembrolizumab (10 mpk) compared to single treatment of amivantamab (*P* < 0.05) and pembrolizumab (*P* < 0.001, *n* = 10 in each group). **G,** Survival curve of LUSC PDX demonstrating improved survival by the combination treatment.

Synergistic benefits of amivantamab and pembrolizumab combination treatment compared to monotherapy were observed in the *in vivo* experiment of HNSCC (YHIM-3003) and LUSC (YHIM-2010) tumor bearing patient derived xenograft (PDX) models. Multiplex IHC of HNSCC and LUSC tumors demonstrated that these models express moderate-to-high levels of EGFR and MET in the TME (Fig. 1A). In the HNSCC PDX, combination of amivantamab and pembrolizumab treatment showed a significant reduction of tumor volume compared to vehicle or single-arm treatments (*P* < 0.001; Fig. 1B). The greatest tumor growth inhibition (TGI) rate was observed largely in the combination treated group (Fig. 1C). TGI of more than 40% was observed in 70% of combination group whereas only 20% of amivantamab group was greater than 40% TGI (Fig. 1C). TGI did not exceed more than 40% in the pembrolizumab group. Additionally, longer survival was observed for the combination treatment group compared to the monotherapy groups. At the end of day 19, 40% of combination group had survived in contrast to pembrolizumab and amivantamab monotherapy where all the mice demised at day 17 and 19, respectively (Fig. 1D).

In the LUSC PDX, amivantamab administered at the same dosage of 30 mpk in combination with pembrolizumab significantly reduced tumor volume compared to pembrolizumab group (*P* < 0.001; Fig. 1E). Interestingly, no rebound of tumor growth was observed in amivantamab and combination group after cessation of drug administration at day 47 (Fig. 1E). It appeared that amivantamab boosted immune activity of lymphocytes including memory phenotypic subsets of T cells and prevented recurrence of tumor development after the treatment has been terminated. However, amivantamab at 30 mpk alone effectively reduced tumor volume (Fig. 1E). This indicated that the dose of amivantamab needed to be decreased in order to evaluate the combination synergy with pembrolizumab. Amivantamab given at a modified dosage of 10 mpk in combination with pembrolizumab significantly reduced the tumor volume compared to amivantamab and pembrolizumab monotherapy groups (*P* < 0.05 and *P* < 0.001, respectively, Fig. 1F). Combination treatment also resulted in improved survival of more than 61 days. All mice in the vehicle and pembrolizumab group demised at day 47 and 53, respectively, where 70% and 40% survived until day 61 in the combination and amivantamab group, respectively (Fig. 1G).

### Combination treatment induced significantly higher proportions of tumor infiltrating CD8^+^ T cells in the TME

**Figure 2:**
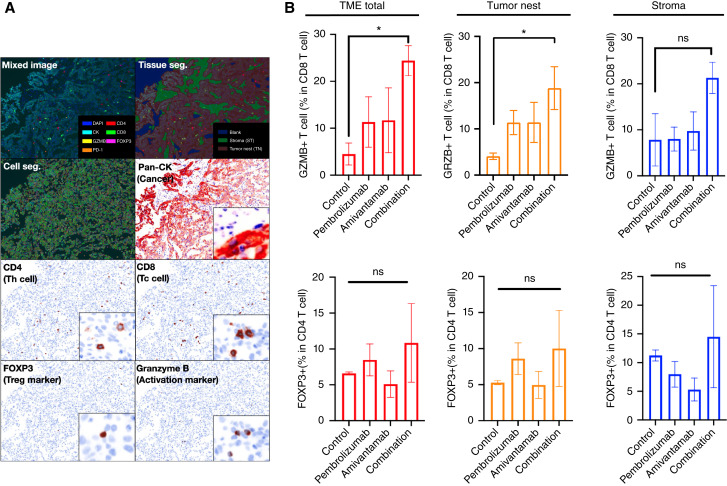
Multiplex IHC showing T cell subpopulations in the tumor microenvironment of HNSCC PDX (YHIM-3003) tumor after combination treatment of amivantamab and pembrolizumab. **A,** Whole slide images of tumor scan showing segmentation of tissues and cells. Helper CD4 and cytotoxic CD8 T cells were marked with FoxP3 (regulatory T cells) and GZMB (active cytotoxic T cells). Cancer cells were stained with Pan-CK. **B,** Stained whole slide images were quantified and presented in bar plots showing GZMB^+^ CD8 T cells and regulatory T cells in the tumor microenvironment, tumor nest and stroma in different treatment groups. Proportion of GZMB^+^ CD8 T cells in the total TME and the tumor nest was significantly increased in the combination group (*P* < 0.05). Each bar in the bar plot represents five mice that were internally sacrificed.

Landscape of the TME within the HNSCC PDX tumor revealed that the infiltration of immune cells in the tumor nest and stromal sites was enhanced by combination treatment of amivantamab and pembrolizumab (Fig. 2A). Whole slide images of the tumor in the treatment groups and the process flow of analysis are shown in the Supplementary Data S1. Proportion of granzyme B (GZMB)-expressing CD8 T cells in tumor nest of TME was significantly increased in the combination group compared to the control group (18.81 ± 10.36 and 4.09 ± 1.55, respectively, *P* < 0.05; Fig. 2B). There were no statistical differences between the groups in the stroma, however, an increasing trend was observed in the combination group. In general, GZMB^+^ CD8^+^ T-cell population increased (in the total area of TME) by combination treatment of amivantamab and pembrolizumab in HNSCC PDX. In contrast, no statistically significant results or trends were observed in the proportion of Foxp3-expressing regulatory CD4^+^ T cells between the treatment groups. These results suggested that amivantamab and pembrolizumab synergistically enhanced infiltration of active cytotoxic CD8^+^ T cells into the TME and suppressed growth of tumor cells.

### Combination treatment induced local and systemic antitumor phenotypic changes in the T-cell populations

**Figure 3:**
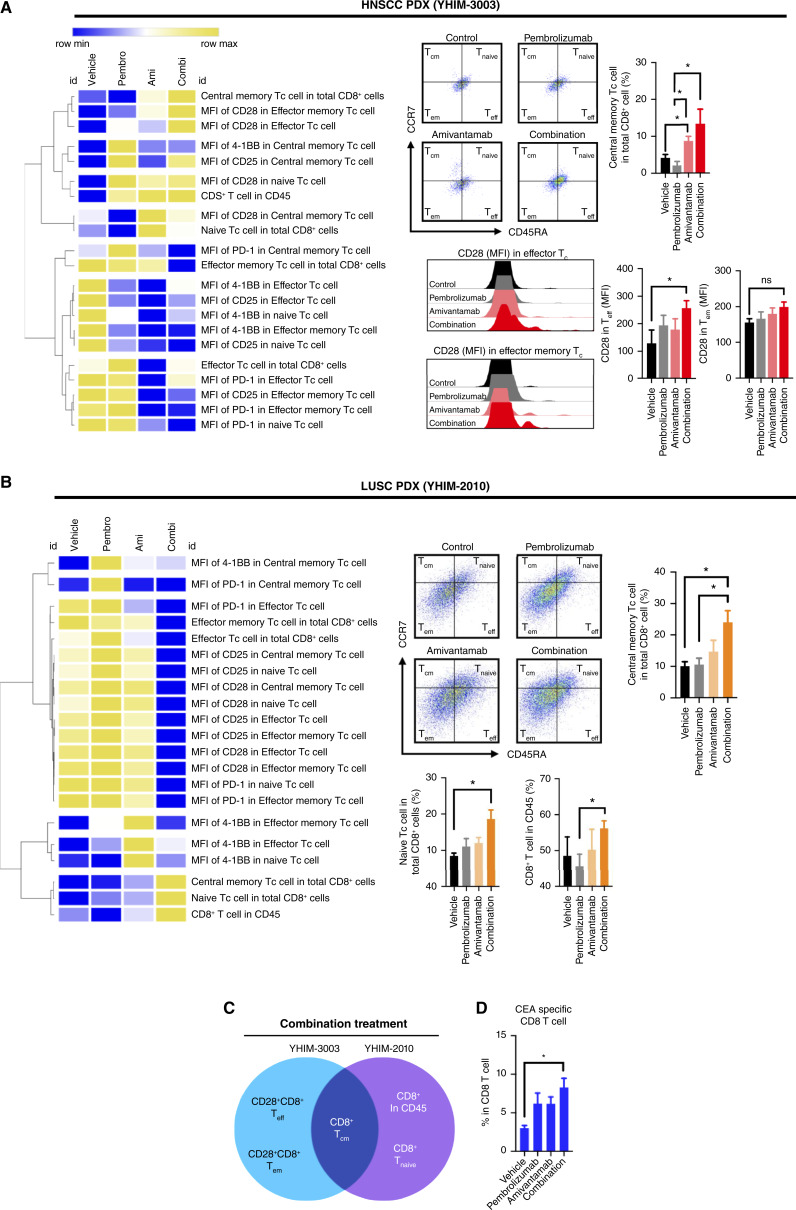
Flow cytometry analysis of memory subsets of T cells in the different treatment groups of humanized HNSCC (YHIM-3003) and LUSC (YHIM-2010) PDX tumor of internally sacrificed mice (*n* = 5 in each treatment group). **A,** Heatmap of memory T cell subsets (central memory, effector memory, and effector T cells) and activation markers in the tumor samples of HNSCC PDX. **B,** Heatmap of memory T-cell subsets (central memory, effector memory, and effector T cells) and activation markers in the tumor samples of YHIM-2010. **C,** Factors that combination of amivantamab and pembrolizumab positively affected in each humanized PDX model and both models shared enhancement of CD8^+^ T central memory subset by combination therapy. **D,** Tumor reactive (CEA-stained) CD8 T cells in HNSCC PDX tumor were abundant in the combination treatment group and were significantly higher in proportion compared to the control group (8.28 ± 2.67 and 3.02 ± 0.75, respectively, *P* < 0.05).

To compare the changes in the populations of lymphocytes between the treatment groups, phenotypic alterations of immune cells were analyzed using flow cytometry. Gating strategies for NK cells and T-cell subsets are described in Supplementary Fig. S2A and S2B, respectively, and myeloid cells in Supplementary Fig. S3. Combination treatment group had significantly increased proportion of central memory CD8^+^ T cells in the TME compared to the single treatment of pembrolizumab in HNSCC PDX (13.38 ± 8.80 and 2.11 ± 2.34 respectively, *P* < 0.05; Fig. 3A) and LUSC PDX (24.00 ± 8.26 and 10.55 ± 4.47 respectively, *P* < 0.05; Fig. 3B). In the HNSCC model, the combination treatment enhanced activity in effector subsets of T cells, showing increased expression of CD28, a costimulatory marker for T-cell activation and survival. Analysis of T cells identified a shared trend between the HNSCC and LUSC PDX models in the central memory subset of cytotoxic CD8^+^ T cells (Fig. 3C). No recurrence of tumor development was observed after the treatment of amivantamab at high dosage in combination with pembrolizumab (Fig. 1E). This observation initially suggested that combination treatment may stimulate memory subsets of T cells during immune response to tumor development. Immune profiling of humanized PDX tumor indicated that combination of amivantamab and pembrolizumab enhanced induction of memory subpopulations of T cells (i.e., central memory CD8^+^ T cells; Fig. 3).

Spleen of PDX mice were processed and stained with tetramer antibodies to detect T lymphocytes that can recognize the tumor specific antigens CEA (carcinoembryonic antigen) that are circulating in the immune system. In HNSCC model, the combination group had significantly higher population of CEA-expressing tumor reactive CD8^+^ T cells compared to the vehicle group (8.28 ± 2.67 and 3.02 ± 0.75, respectively, *P* < 0.05; Fig. 3D). The combination group also had the highest proportion compared to amivantamab (6.17 ± 1.98) and pembrolizumab (6.20 ± 3.05; Fig. 3D). No significant differences were observed in the LUSC model.

### Pembrolizumab monotherapy induces an EGFR^HIGH^MET^HIGH^ subcluster that upregulates genes implicated in lactate production and immune suppression

**Figure 4:**
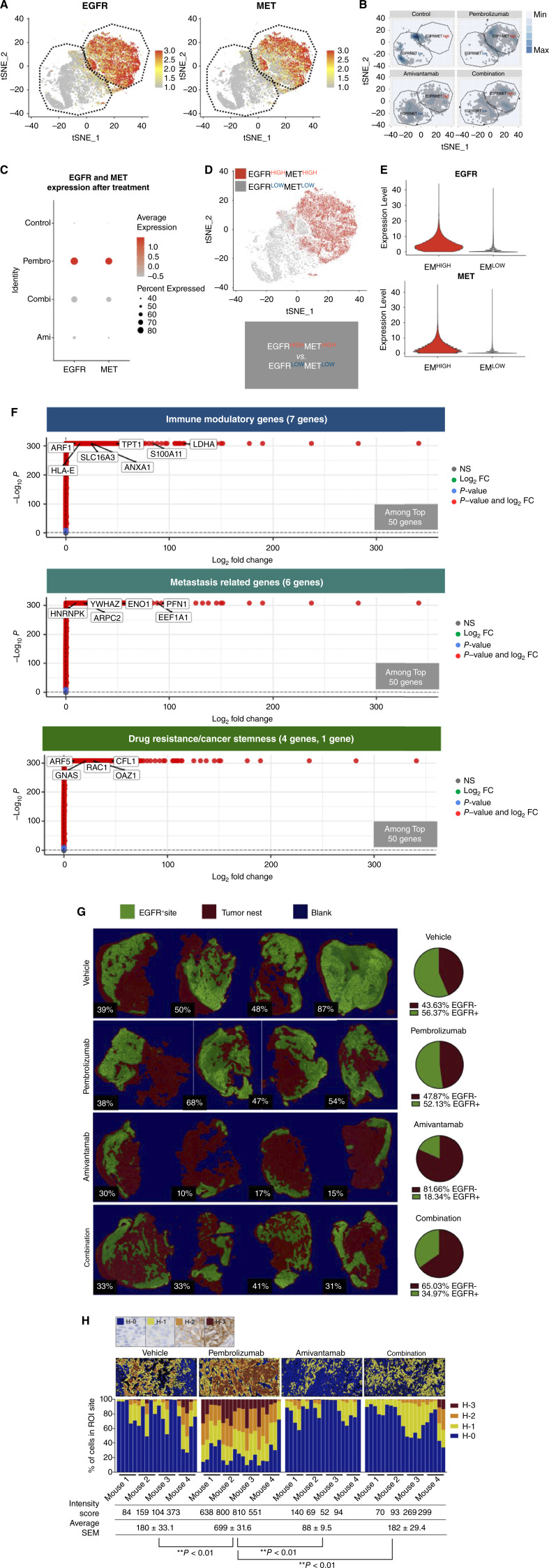
Single-cell RNA sequencing analysis of EGFR^HIGH^/MET^HIGH^ and EGFR^LOW^/MET^LOW^ subclusters in the tumor of humanized HNSCC (YHIM-3003) PDX mice showing relative increase in EGFR and MET expressing subcluster after treatment of pembrolizumab (*n* = 5 in each treatment group). Top 50 genes were analyzed by log_2_ fold change (FC) against *P*-values and immune related genes were divided into three categories as following: immunomodulation, metastasis potential and cancer progression (drug resistance/cancer stemness). **A,** Heatmap of tumor indicating a region of tumor subcluster with elevated expression of both EGFR and MET. **B,** Density plot illustrating EGFR^HIGH^/MET^HIGH^ and EGFR^LOW^/MET^LOW^ subclusters in treatment groups. Expression of EGFR and MET was relatively higher in the pembrolizumab treated group compared to the other treatment groups (**C**). **D,** Tumor subcluster with elevated dual expression of EGFR and MET (EM^HIGH^) was define and analyzed for DEGs. **E,** EGFR and MET in EM^HIGH^ and EM^LOW^, showing increased expression of both markers in EM^HIGH^ tumor cluster. **F,** DEG analysis of the top genes in the EGFR^HIGH^/MET^HIGH^ tumor subcluster showing genes related to immunomodulation, tumor metastasis, drug resistance and cancer stemness in volcano plot. **G,** Multiplex IHC of the tumor tissue showing reduced expression of EGFR in tumor treated with combination treatment. EGFR+ site, colored green, represents tumor regions that were stained positive for EGFR and does not directly translate to the level of expression. Tumor with damaged or indistinct regions of tumor nest was treated as an outlier and was removed from each group. **H,** Fluorescence intensity of EGFR in randomized region of interest sites converted into average H-scores. H-scores ranged from 0 (H-0, blue) to 3 (H-3, red), with H-3 representing the highest intensity as shown in the top. Average *H*-score of EGFR expression in pembrolizumab treated mice was significantly higher than the other groups (*P* < 0.01).

Single-cell RNA sequencing analysis of the tumor was performed to further investigate the immunomodulatory effects of combination therapy and compare the changes in expression of biomarkers between the treatment groups. In HNSCC PDX tumor, a subcluster with high expression of EGFR simultaneously had elevated level of MET (Fig. 4A). Interestingly, single treatment of pembrolizumab induced higher density of EGFR and MET dual expressing subcluster compared to the other treatment groups (Fig. 4B and C). The tumor subcluster expressing high levels of both EGFR and MET (EM^HIGH^) was define and analyzed for top 50 genes to identify differentially expressed genes (DEGs) in EGFR^HIGH^/MET^HIGH^ and EGFR^LOW^/MET^LOW^ tumor subclusters (Fig. 4D–F). Among the top genes, ANXA1, ARF1, HLA-E, lactate dehydrogenase A (LDHA), SLC16A3, S100A11, and TPT1 were key immunomodulatory factors that showed significant fold changes, with LDHA and SLC16A3 showing 128 and 25 log fold changes, respectively (*P* < 0.05 with log_2_ fold change ≥ 1, Fig. 4F), indicating that these markers were significantly elevated in the EGFR^HIGH^/MET^HIGH^ subcluster compared to the EGFR^LOW^/MET^LOW^ subcluster. These immune modulatory markers were also key factors in the EGFR^HIGH^ subcluster of the LUSC PDX tumor (Supplementary Fig. S4A and S4B). In this study, LDHA and SLC16A3 were revealed as core genes with regulatory functions in the biological process of glucose metabolism that may hinder immune surveillance and responses within the TME. It appeared that tumor sites with positive staining of EGFR were comparable between the vehicle and pembrolizumab groups, while EGFR^+^ sites were less abundant in amivantamab and combination treated groups (Fig. 4G). However, the proportion of tumor cells with higher intensity of EGFR expression was significantly greater in the pembrolizumab group (Fig. 4H). Intensity score of EGFR in the pembrolizumab group was 699 ± 31.6, which was significantly higher than the score of vehicle, amivantamab, and combination group (180 ± 33.1, 88 ± 9.5, 182 ± 29.4, respectively, *P* < 0.01; Fig. 4H). This suggested that a large proportion of the tumor actively upregulated the expression of EGFR and MET in one of multifaceted responses to pembrolizumab. It is feasible that upregulation of EGFR and MET in tumor cells contributed to resistance to pembrolizumab monotherapy (Fig. 1B).

### Amivantamab downregulates genes induced by EGFR/MET high subcluster and promotes antitumor immunity

**Figure 5:**
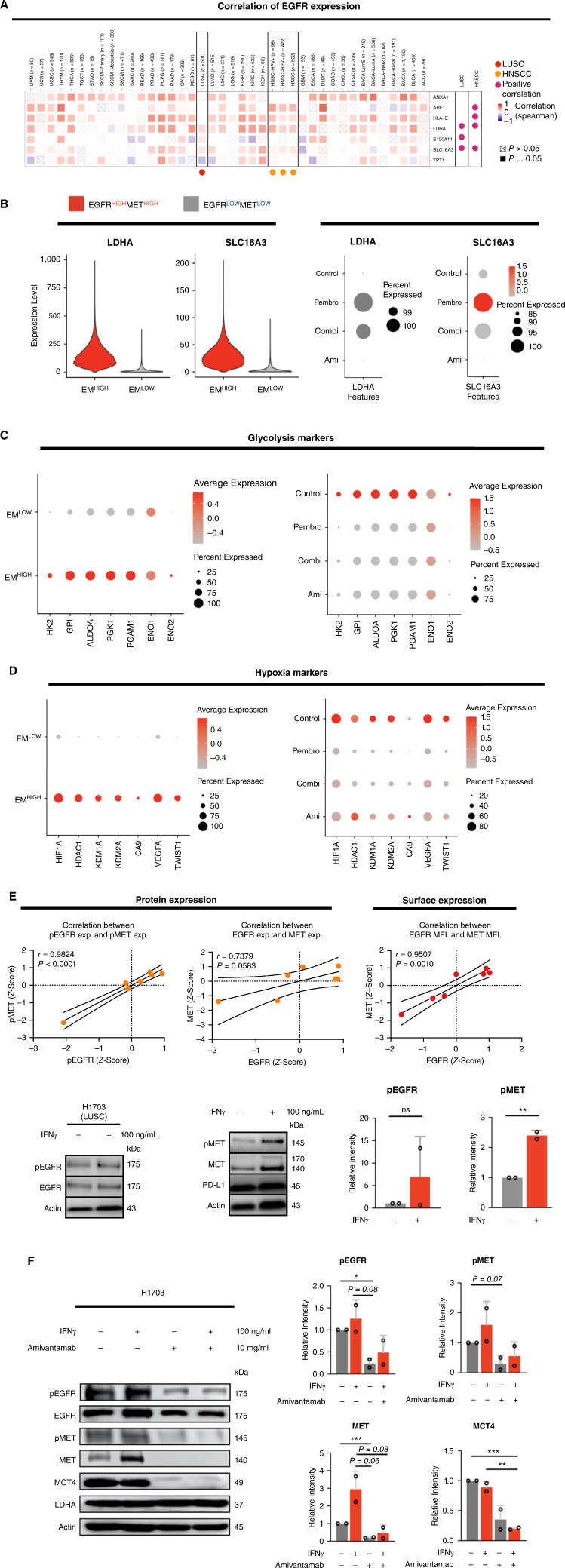
LDHA and SLC16A3 are important regulators of glycolytic pathways and were upregulated in EGFR^HIGH^/MET^HIGH^ tumor subcluster of HNSCC PDX (YHIM-3003). **A,** Correlation between expression of EGFR and biomarkers based on TCGA database. LDHA and SLC16A3 expression was positively correlated with expression of EGFR in both HNSCC and LUSC. **B,** Expression of LDHA and SLC16A3 was significantly increased in EGFR^HIGH^/MET^HIGH^ tumor subcluster (EM^HIGH^) of HNSCC PDX (left). Additionally, expression of LDHA and SLC16A3 increased in pembrolizumab treated group. **C,** Regulators of glycolysis (HK2, GPI, ALDOA, PGK1, PGAM1, ENO1, ENO2) comparatively increased in the EM^HIGH^ tumor subcluster (left) and pembrolizumab treated group (right). **D,** Regulators of hypoxia (HIF1A, HDAC1, KDM1A, KDM2A) and downstream signaling markers (CA9, VEGFA, TWIST1) increased in the EM^HIGH^ tumor subcluster (left) and pembrolizumab treated group (right). **E,** Total protein and surface expression of EGFR and MET demonstrated strong correlation in HNSCC and LUSC cell lines (left). H1703, LUSC human cancer cell, was treated with IFNγ for 24 hours to mimic the physiological response of pembrolizumab in the TME (right). Induction of IFNγ, though not significant, upregulated the expression of pEGFR, while pMET level significantly increased (*P* < 0.01). **F,** Protein expression of EGFR/p-EGFR, MET/p-MET, MCT4 (SLC16A3), and LDHA after 72 hours of amivantamab at 10 mg/mL in H1703. IFNγ was treated at 100 ng/mL for 24 hours (left). Amivantamab reduced the expression of these genes under IFNγ-induced conditions (right).

Based on the public database of The Cancer Genome Atlas (TCGA), expression of EGFR in both HNSCC and LUSC significantly correlated with the expression of LDHA and SLC16A3 (*P* < 0.05; Fig. 5A), suggesting that regulation of EGFR and glucose metabolism simultaneously contribute to TME favoring progression of tumor. In this study, expression of LDHA and SLC16A3 was significantly higher in the EGFR^HIGH^/MET^HIGH^ subcluster of HNSCC PDX tumor compared to the EGFR^LOW^/MET^LOW^ subcluster (*P* < 0.05; Fig. 5B). Additionally, the expression of LDHA and SLC16A3 was notably higher in the pembrolizumab group (Fig. 5B). Transcriptional upregulation of other glycolytic markers was also predominantly increased in the EGFR^HIGH^/MET^HIGH^ subcluster and the pembrolizumab group (HK2, GPI, ALDOA, PGK1, PGAM1, ENO1, and ENO2; Fig. 5C). Moreover, key regulators of hypoxia showed the same trend in the EGFR^HIGH^/MET^HIGH^ subcluster and the treatment groups (HIF1A, HDAC1, KDM1A, KDM2A, CA9, VEGFA, and TWIST1; Fig. 5D), suggesting that the tumor in the HNSCC PDX mice treated with pembrolizumab feasibly had an acidic environment by favoring anaerobic metabolism of glucose in the TME. Similar trend in the expression changes of these key markers was observed in the LUSC PDX model (Supplementary Fig. S4C and S4D).

Correlation of translational and surface expression of EGFR and MET was also confirmed in HNSCC and LUSC cell lines without EGFR mutations (Supplementary Fig. S5A). T cells secrete interferon gamma (IFNγ) that functions as autocrine/paracrine molecule in response to pembrolizumab in the tumor site. Thus, cell lines were treated with IFNγ to replicate the immunological stimulus in the TME of PDX models by treatment of pembrolizumab. At the protein level, upregulation of EGFR and MET in selected HNSCC and LUSC cell lines after IFNγ induction showed a moderate correlation (Supplementary Fig. S5B), while expression of pEGFR and pMET manifested a strong positive correlation (*r* = 0.9824, *P* < 0.0001; Fig. 5E). In addition, IFNγ-induced surface expression of EGFR and MET showed positive correlation (*r* = 0.9507, *P* < 0.005; Fig. 5E). Induction of IFNγ increased expression of pEGFR and pMET (*P* < 0.01) in H1703, indicating that IFNγ initiates signaling cascade of EGFR and MET. In general, expression of EGFR and MET and downstream signaling molecules was inhibited by amivantamab with or without the presence of IFNγ (Fig. 5F). Importantly, amivantamab significantly reduced the expression of SLC16A3 (monocarboxylate transporter 4, MCT4), one of the key regulators of glycolysis identified by single-cell RNA sequencing analysis in humanized PDX models, in the presence of IFNγ (*P* < 0.01; Fig. 5F).

### Amivantamab also reduced immune checkpoint–related markers including PD-L1 in the EGFR^HIGH^MET^HIGH^ tumor subcluster

**Figure 6:**
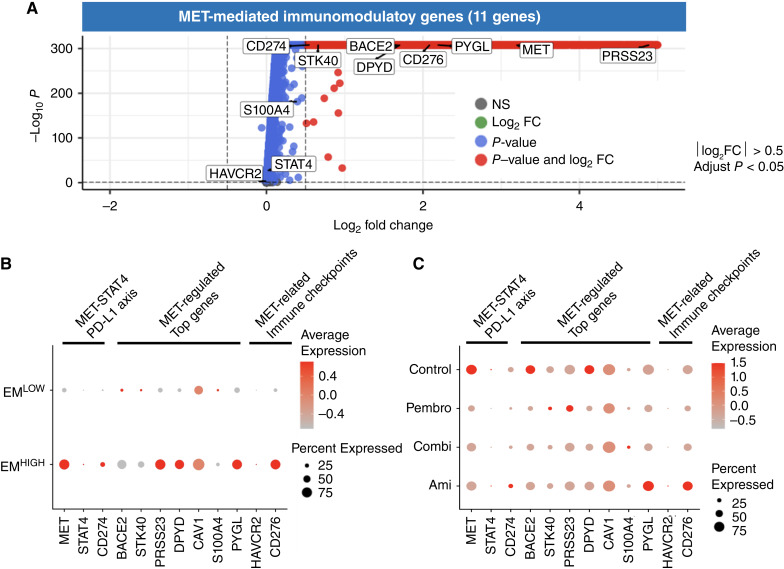
Upregulation of EGFR and MET in HNSCC PDX (YHIM-3003) tumor induced increased expression of immune checkpoints regulators in the EGFR^HIGH^/MET^HIGH^ subcluster (EM^HIGH^). **A,** Volcano plot of top 50 genes in EGFR^HIGH^/MET^HIGH^ against EGFR^LOW^/MET^LOW^ tumor subcluster analyzed by log2 fold change (FC) against *P*-values. Red dots indicating transcripts with significantly increased fold changes including MET, PD-L1, and MET-regulated genes. **B,** Expression of STAT-4/PD-L1 (MET, STAT4, CD274), MET-regulated (BACE2, STK40, PRSS23, DPYD, CAV1, S100A4, PYGL), and MET-related immune checkpoints (HAVCR2, CD276) generally increased in the EGFR^HIGH^/MET^HIGH^ tumor subcluster compared to the EGFR^LOW^/MET^LOW^ subcluster. **C,** Expression of MET-related markers in different treatment groups.

DEG analysis of HNSCC PDX tumor illustrated that MET-regulated genes and other immune checkpoint markers were also increased in the EGFR^HIGH^/MET^HIGH^ tumor subcluster. Significantly increased MET-related genes were BACE2, CD274, CD276, DPYD, PRSS23, PYGL, STK40, and S100A4 (Fig. 6A). In addition to elevated MET-regulated genes, MET-STAT4-PD-L1 axis had noticeably increased expression in the EGFR^HIGH^/MET^HIGH^ tumor group (Fig. 6B). It appeared that expression of these markers had decreased in the combination group, suggesting that amivantamab and pembrolizumab combination impeded downstream signaling of MET-regulated genes (Fig. 6C; Supplementary Fig. S4E and S4F).

## Discussion

Our study demonstrated combinatorial benefits of amivantamab and pembrolizumab by effectively remodeling the tumor-immune microenvironment, providing a strong preclinical rationale to clinically combine amivantamab and PD-1 blockade treatment. In fact, there is an ongoing study of combination therapy with amivantamab and cetrelimab in patients with metastatic NSCLC (PolyDamas, NCT05908734), and the clinical results are awaited.

The combination synergy of amivantamab and pembrolizumab was observed in the treatment of humanized PDX mouse model bearing, pembrolizumab-insensitive HSNCC (YHIM-3003) and LUSC (YHIM-2010) tumors. By comprehensive analysis of immunogenic mechanism behind the combination synergy, we noted that the combination treatment enhanced stimulation of central memory subset of cytotoxic CD8^+^ T cells in both PDX models, and induced tumor-reactive T cells in HNSCC PDX. Stimulation of memory subsets may help to understand why there was no rebound of tumor after the treatment had stopped (Fig. 1E). Central memory subset of CD8^+^ T cells have shown superior *in vivo* and *in vitro* antitumor immunity compared with effector memory T cells (19–21). In addition to modulating T cells, combination treatment increased proportion of GZMB-expressing cytotoxic CD8^+^ T cells in the TME, suggesting that combination treatment encouraged infiltration of physiologically active CD8^+^ T cells into the tumor nest. These data suggest that combination treatment can ultimately enhance T-cell-mediated tumor killing in the tumors that were previously nonresponsive to immunotherapy.

We subsequently analyzed the single-cell transcriptomic landscape of the tumor and revealed that tumor cells exhibited relatively high EGFR and MET expression in response to pembrolizumab treatment alone. EGFR signaling in cancers has been associated with global metabolism favoring highly glycolytic tumors (22). We demonstrated that EGFR^HIGH^ tumor subcluster was distinctly elevated in the pembrolizumab-treated group and exhibited significantly higher level of lactate producing biomarkers including LDHA and SLC16A3 compared to the combination group in both HNSCC and LUSC PDX. Our findings suggest that pembrolizumab may promote a tumor-immune microenvironment with metabolic characteristics of the “Warburg phenotype” in the tumor (23, 24). LDHA is essential for conversion of pyruvate into lactate while SLC16A3 facilitates exchange of lactate between the cells and extracellular matrix (ECM). It is likely that the EGFR^HIGH^ tumor cells produced and actively exported lactate into the ECM of TME. Lactate provides metabolic fuel for cancer cells as well as tumor-killing immune cells such as T and NK cells, however, accumulation of lactate due to enhanced glycolysis greatly disables the ability of CD8^+^ T and NK cells to infiltrate into the tumor site (25–27). Several studies have also highlighted that lactate suppressed cytotoxic activity of CD8^+^ T cells by inhibiting production of IFNγ, which is crucial for facilitating tumor killing by CD8^+^ T and NK cells (28–30). This suggests that the tumor with high EGFR expression may be unresponsive to immunotherapy by disturbing essential mechanism and subsequent loss of functionality in cytotoxic T and NK cells in the TME (31). In fact, the single-agent activity of pembrolizumab alone in HNSCC, which expresses high levels of EGFR and MET, has an objective response rate of 16.9%, with the median progression-free survival of 2.3 months (32).

In addition to creating metabolic environment that favors tumor persistence, we found that pembrolizumab treatment also increased MET-related immune checkpoint markers and expression of PD-L1. It appeared that pembrolizumab treatment alone induced upregulation of immune checkpoint in the tumor cells, contributing to becoming more resistant to T cell killing and evasion of the immune response via enhanced signaling through MET-STAT4-PD-L1 axis (33, 34). We hypothesize that the HNSCC PDX model became insensitive to anti-PD-1 immunotherapy alone by developing multifaceted bypassing mechanisms, upregulating MET signaling pathway in addition to amplification of EGFR. Our data suggest that amivantamab diminishes the immunosuppressive effects by pembrolizumab on the immune cells in the TME. Significant reduction of glycolytic and MET-regulated immune checkpoint biomarkers in the combination treatment group restored infiltration and activation of infiltrating cytotoxic CD8^+^ T and NK cells, which was also evident in the *in vivo* models and comprehensive immune profiling analysis. These phenomena were less evidently seen in the LUSC model, as there was less abundant co-expression of EGFR and MET in the tumor. However, gene expression analysis showed similar trend in the EGFR and MET.

Upon binding with major histocompatibility complex molecules, T cells secrete IFN-γ to enhance antitumor activity of surrounding T lymphocytes (21). IFNγ signaling also promotes MET activation and induce immune checkpoints via enhanced MET-STAT4-PD-L1 axis in tumor cells, providing tumor cells with immune evasion mechanism (33, 35, 36). This was also evident in our study that MET-STAT4-PD-L1 axis and MET-related immune checkpoints were elevated particularly in the pembrolizumab-treated EGFR^HIGH^ tumor. These alterations are likely to facilitate tumor growth by allowing immune tolerance and may affect the response to immune checkpoint inhibitors.

Immunological and molecular changes were evident in the HNSCC PDX tumor throughout this study. However, there were some inconclusive observations and limitations in the LUSC PDX model due to inherent heterogeneous characteristics of the tumor. The tumor of LUSC PDX mice generally had low abundance and infiltration of immune cells in the TME, deemed as “cold tumor,” and also exhibited high intraspecific and interspecific variations between the samples, particularly in the multiplex IHC analysis of the TME. The molecular analysis of LUSC model appeared statistically significant and similar changes were observed in phenotypic changes of T cells and expression profile of EGFR^HIGH^ tumor subcluster. It may be that pretreatment of tumor samples allowed extraction of T lymphocytes during rigorous dissociation process and enabled profiling and expression analysis of immune cells. However, the physical contour of the tumor nest was inconsistent and led to poor visualization of the TME using multiplex IHC, suggesting that an earlier timepoint may have provided a better representation of the LUSC PDX model. Another limitation of this study is that it would have benefited from knockout or overexpression assay followed by assessment of lactate levels and further validate the subsequent downstream effects on the activity of T cells in *in vivo* and *in vitro*.

In conclusion, we revealed that amivantamab diminished the immunosuppressive effects induced by pembrolizumab in the EGFR^HIGH^MET^HIGH^ tumor of pembrolizumab insensitive humanized HNSCC PDX model. Combination of amivantamab and pembrolizumab significantly reduced tumor volume in HNSCC and LUSC tumor–bearing PDX model compared to amivantamab or pembrolizumab alone. We demonstrated that combination treatment greatly enhanced antitumor phenotypic changes in infiltrating CD8^+^ T cells and central memory T cells in the TME. Single-cell RNA transcriptomic analysis showed that pembrolizumab alone induced tumor invasive mechanism by upregulation of EGFR and MET signaling. However, the same biomarkers were reduced when pembrolizumab was administered in combination with amivantamab. This study highlighted rationale for combination therapy of amivantamab and PD-1 blockade immunotherapy that can be applied in clinical treatment regimen of solid advanced tumors.

## Supplementary Material

Supplementary Figure 1 This figure entails the selection of HNSCC and LUSC PDX model based on the H-score of EGFR and MET.

Supplementary Figure 2 Figure S2 shows the gating strategy for T panel in immune profiling analysis.

Supplementary Figure 3 Figure S3 shows the gating strategy for M panel in immune profiling analysis.

Supplementary Figure 4 This figure shows the single cell RNA sequencing analysis of EGFR high tumor subcluster in the humanized LUSC PDX model.

Supplementary Figure 5 This figure shows the selection of cell lines used for in vitro evaluation of the effects of amivantamab on the protein and surface levels EGFR, MET, LDHA and SLC16A3.

Supplementary Table 1 This table entails staining panels of antibodies used for flow cytometry analysis of PDX tumor samples.

Supplementary Data 1 Legend Data legend for supplementary data 1, showing the process chart and raw whole slide images of multiplex IHC.

Supplementary Data 1 Individual whole slide images of HNSCC PDX tumor used for TME analysis.
