# Supplementary material for: Exploration of Immune-Modulatory Effects of Amivantamab in Combination with Pembrolizumab in Lung and Head and Neck Squamous Cell Carcinoma
Source: Cancer Res Commun. 2024 Jul 17;4(7):1748–64. doi: 10.1158/2767-9764.CRC-24-0107 (PMC11253790; doi:10.1158/2767-9764.CRC-24-0107)
Supplement: Supplementary Figure 1 — This figure entails the selection of HNSCC and LUSC PDX model based on the H-score of EGFR and MET. [file crc-24-0107_supplementary_figure_1_supps1.pptx]

## Slide 1
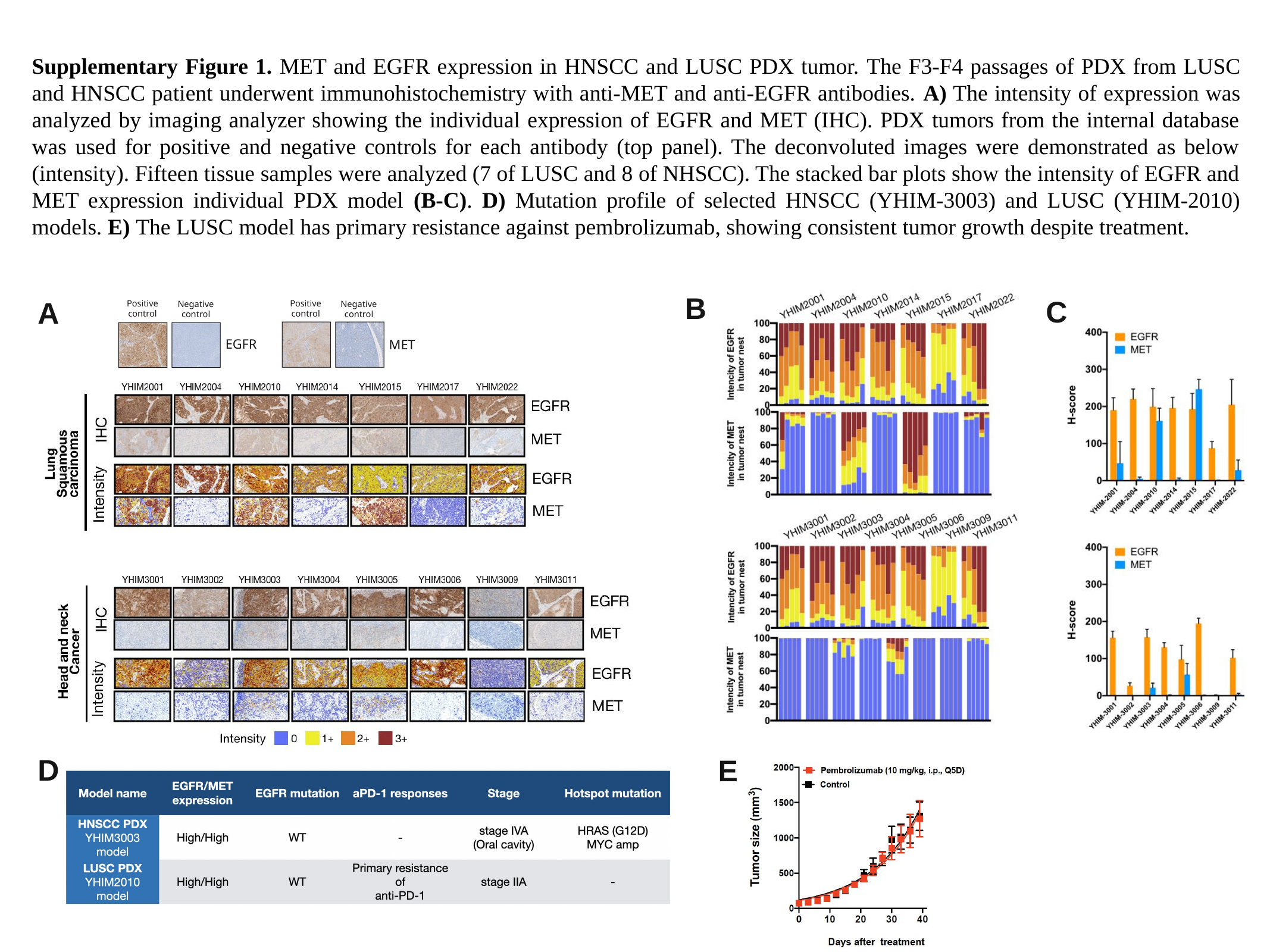

Supplementary Figure 1. MET and EGFR expression in HNSCC and LUSC PDX tumor. The F3-F4 passages of PDX from LUSC and HNSCC patient underwent immunohistochemistry with anti-MET and anti-EGFR antibodies. A) The intensity of expression was analyzed by imaging analyzer showing the individual expression of EGFR and MET (IHC). PDX tumors from the internal database was used for positive and negative controls for each antibody (top panel). The deconvoluted images were demonstrated as below (intensity). Fifteen tissue samples were analyzed (7 of LUSC and 8 of NHSCC). The stacked bar plots show the intensity of EGFR and MET expression individual PDX model (B-C). D) Mutation profile of selected HNSCC (YHIM-3003) and LUSC (YHIM-2010) models. E) The LUSC model has primary resistance against pembrolizumab, showing consistent tumor growth despite treatment.
B
C
A
Positive
control
Positive
control
Negative
control
Negative
control
EGFR
MET
D
E
