# Supplementary material for: Exploration of Immune-Modulatory Effects of Amivantamab in Combination with Pembrolizumab in Lung and Head and Neck Squamous Cell Carcinoma
Source: Cancer Res Commun. 2024 Jul 17;4(7):1748–64. doi: 10.1158/2767-9764.CRC-24-0107 (PMC11253790; doi:10.1158/2767-9764.CRC-24-0107)
Supplement: Supplementary Figure 2 — Figure S2 shows the gating strategy for T panel in immune profiling analysis. [file crc-24-0107_supplementary_figure_2_supps2.pptx]

## Slide 1
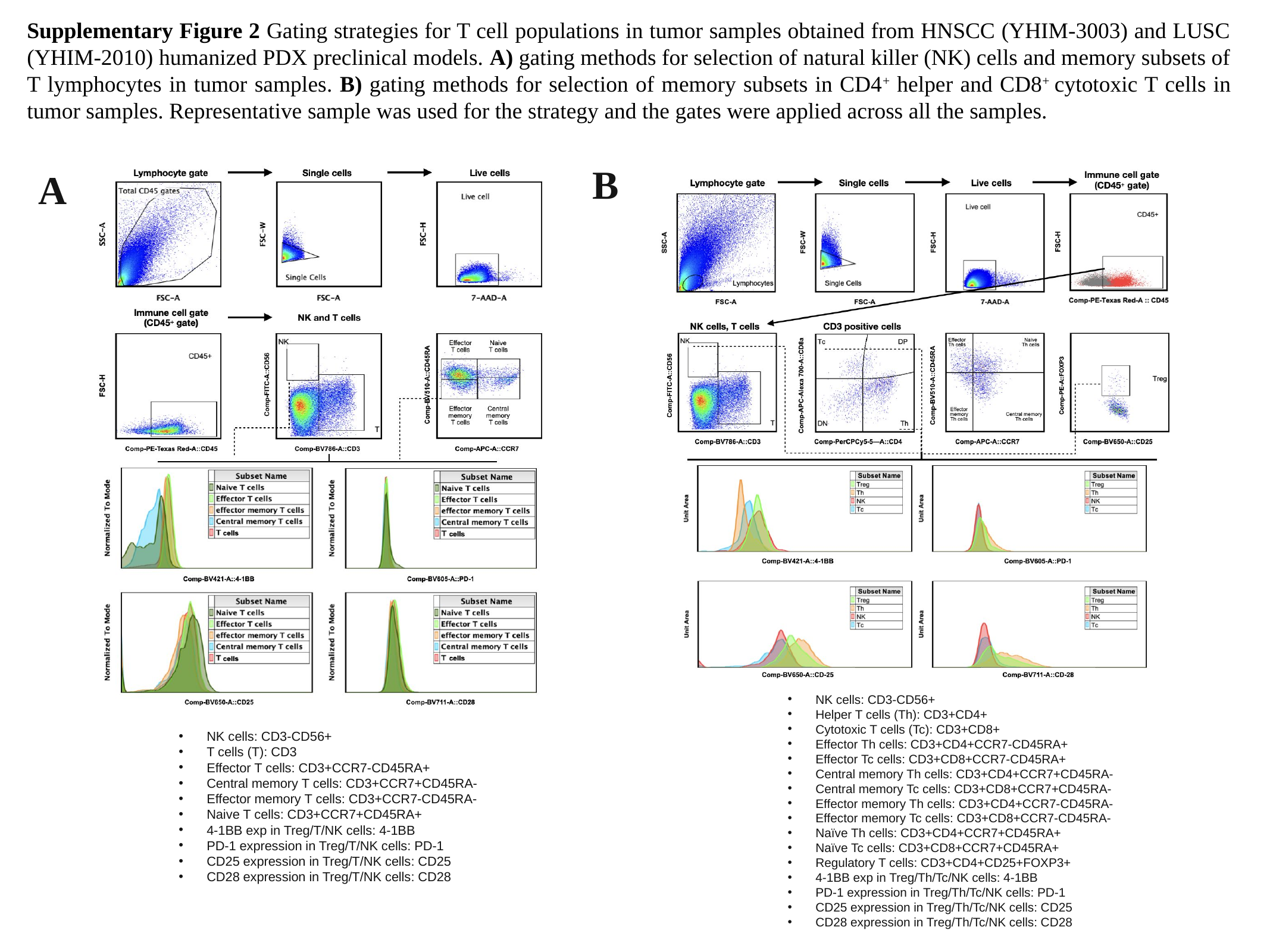

Supplementary Figure 2 Gating strategies for T cell populations in tumor samples obtained from HNSCC (YHIM-3003) and LUSC (YHIM-2010) humanized PDX preclinical models. A) gating methods for selection of natural killer (NK) cells and memory subsets of T lymphocytes in tumor samples. B) gating methods for selection of memory subsets in CD4+ helper and CD8+ cytotoxic T cells in tumor samples. Representative sample was used for the strategy and the gates were applied across all the samples.
B
A
NK cells: CD3-CD56+
Helper T cells (Th): CD3+CD4+
Cytotoxic T cells (Tc): CD3+CD8+
Effector Th cells: CD3+CD4+CCR7-CD45RA+
Effector Tc cells: CD3+CD8+CCR7-CD45RA+
Central memory Th cells: CD3+CD4+CCR7+CD45RA-
Central memory Tc cells: CD3+CD8+CCR7+CD45RA-
Effector memory Th cells: CD3+CD4+CCR7-CD45RA-
Effector memory Tc cells: CD3+CD8+CCR7-CD45RA-
Naïve Th cells: CD3+CD4+CCR7+CD45RA+
Naïve Tc cells: CD3+CD8+CCR7+CD45RA+
Regulatory T cells: CD3+CD4+CD25+FOXP3+
4-1BB exp in Treg/Th/Tc/NK cells: 4-1BB
PD-1 expression in Treg/Th/Tc/NK cells: PD-1
CD25 expression in Treg/Th/Tc/NK cells: CD25
CD28 expression in Treg/Th/Tc/NK cells: CD28
NK cells: CD3-CD56+
T cells (T): CD3
Effector T cells: CD3+CCR7-CD45RA+
Central memory T cells: CD3+CCR7+CD45RA-
Effector memory T cells: CD3+CCR7-CD45RA-
Naive T cells: CD3+CCR7+CD45RA+
4-1BB exp in Treg/T/NK cells: 4-1BB
PD-1 expression in Treg/T/NK cells: PD-1
CD25 expression in Treg/T/NK cells: CD25
CD28 expression in Treg/T/NK cells: CD28
