# Supplementary material for: Exploration of Immune-Modulatory Effects of Amivantamab in Combination with Pembrolizumab in Lung and Head and Neck Squamous Cell Carcinoma
Source: Cancer Res Commun. 2024 Jul 17;4(7):1748–64. doi: 10.1158/2767-9764.CRC-24-0107 (PMC11253790; doi:10.1158/2767-9764.CRC-24-0107)
Supplement: Supplementary Figure 3 — Figure S3 shows the gating strategy for M panel in immune profiling analysis. [file crc-24-0107_supplementary_figure_3_supps3.pptx]

## Slide 1
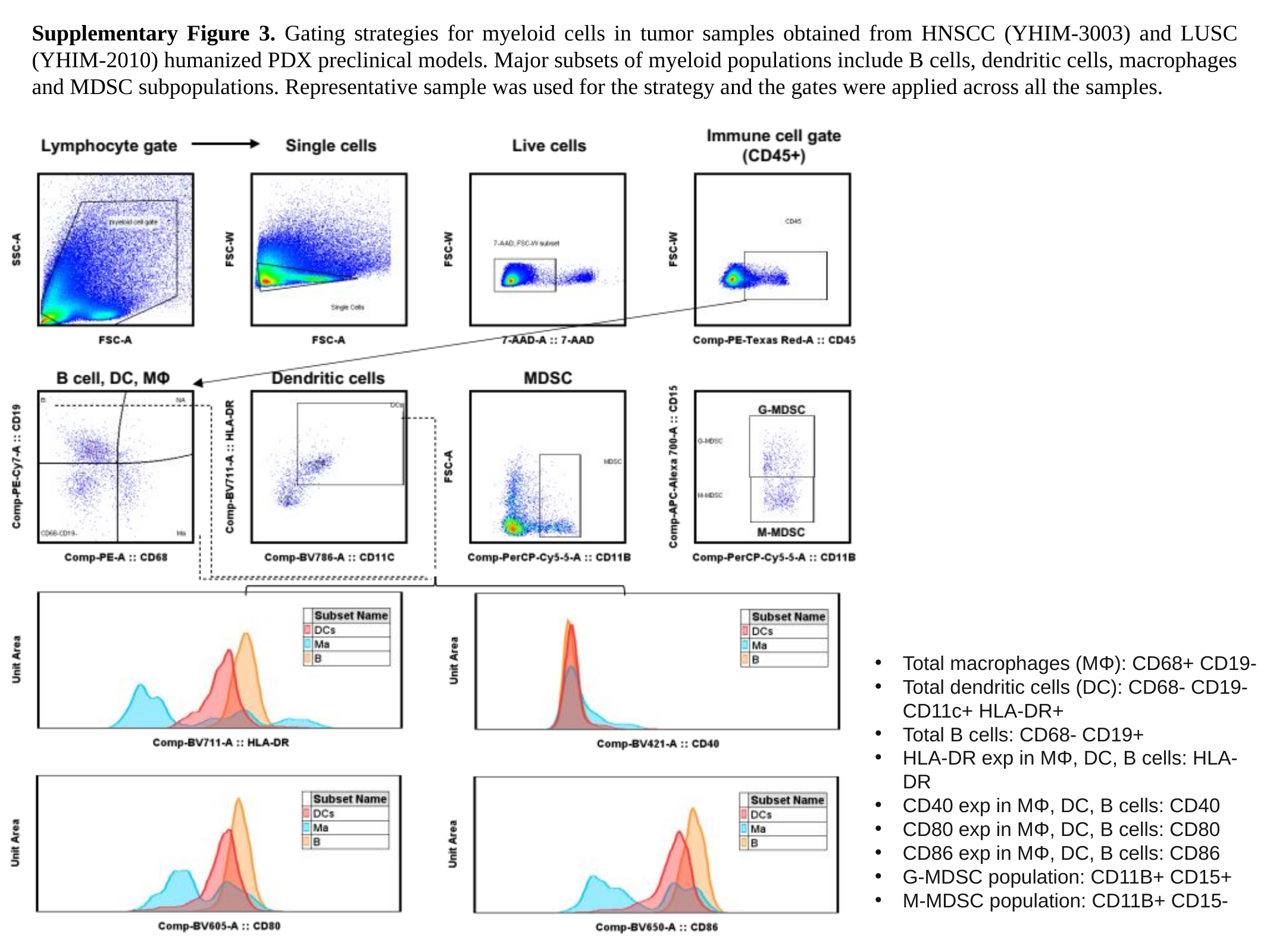

Supplementary Figure 3. Gating strategies for myeloid cells in tumor samples obtained from HNSCC (YHIM-3003) and LUSC (YHIM-2010) humanized PDX preclinical models. Major subsets of myeloid populations include B cells, dendritic cells, macrophages and MDSC subpopulations. Representative sample was used for the strategy and the gates were applied across all the samples.
Total macrophages (MΦ): CD68+ CD19-
Total dendritic cells (DC): CD68- CD19- CD11c+ HLA-DR+
Total B cells: CD68- CD19+
HLA-DR exp in MΦ, DC, B cells: HLA-DR
CD40 exp in MΦ, DC, B cells: CD40
CD80 exp in MΦ, DC, B cells: CD80
CD86 exp in MΦ, DC, B cells: CD86
G-MDSC population: CD11B+ CD15+
M-MDSC population: CD11B+ CD15-
