# Supplementary material for: Exploration of Immune-Modulatory Effects of Amivantamab in Combination with Pembrolizumab in Lung and Head and Neck Squamous Cell Carcinoma
Source: Cancer Res Commun. 2024 Jul 17;4(7):1748–64. doi: 10.1158/2767-9764.CRC-24-0107 (PMC11253790; doi:10.1158/2767-9764.CRC-24-0107)
Supplement: Supplementary Figure 4 — This figure shows the single cell RNA sequencing analysis of EGFR high tumor subcluster in the humanized LUSC PDX model. [file crc-24-0107_supplementary_figure_4_supps4.pptx]

## Slide 1
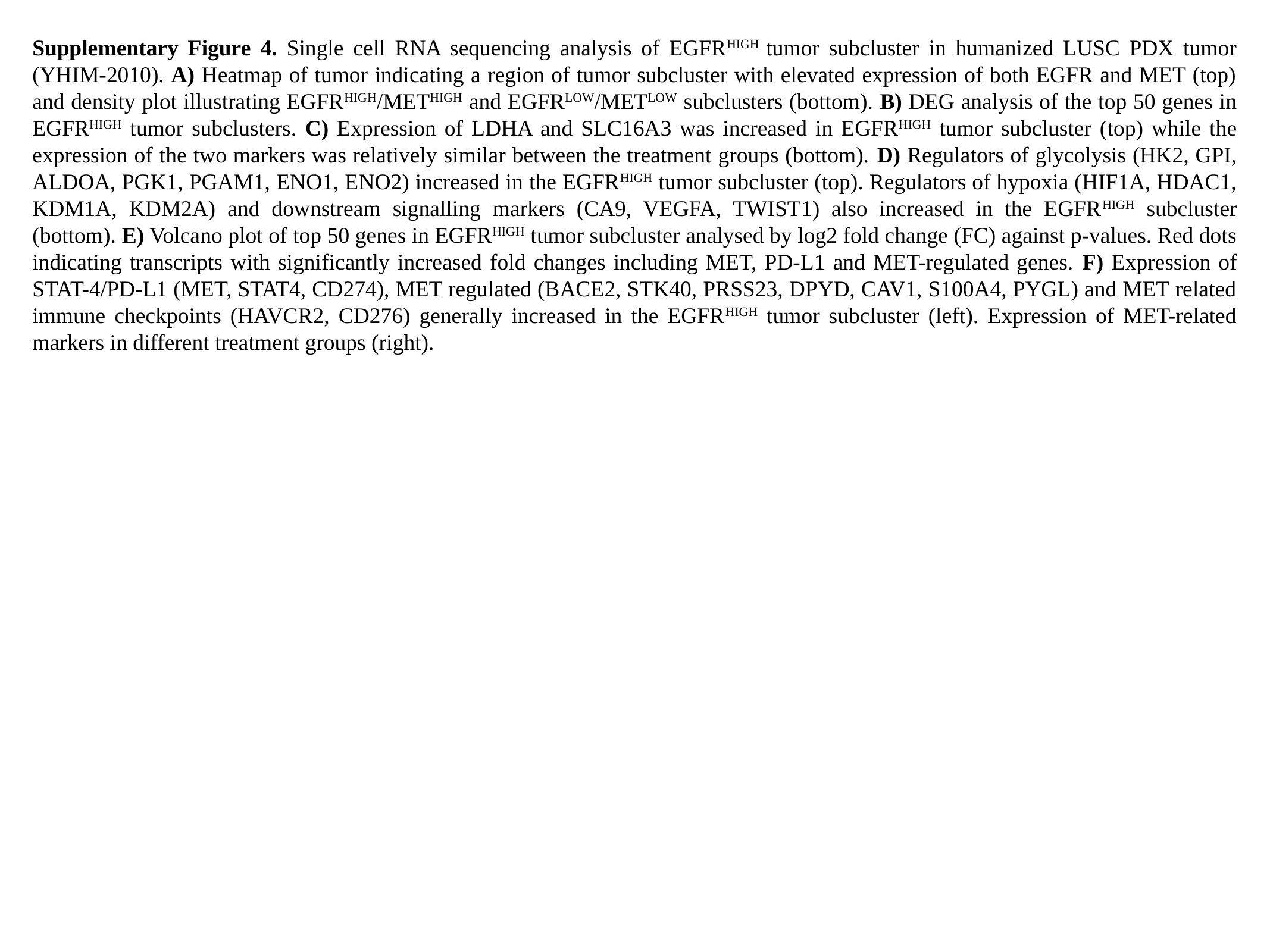

Supplementary Figure 4. Single cell RNA sequencing analysis of EGFRHIGH tumor subcluster in humanized LUSC PDX tumor (YHIM-2010). A) Heatmap of tumor indicating a region of tumor subcluster with elevated expression of both EGFR and MET (top) and density plot illustrating EGFRHIGH/METHIGH and EGFRLOW/METLOW subclusters (bottom). B) DEG analysis of the top 50 genes in EGFRHIGH tumor subclusters. C) Expression of LDHA and SLC16A3 was increased in EGFRHIGH tumor subcluster (top) while the expression of the two markers was relatively similar between the treatment groups (bottom). D) Regulators of glycolysis (HK2, GPI, ALDOA, PGK1, PGAM1, ENO1, ENO2) increased in the EGFRHIGH tumor subcluster (top). Regulators of hypoxia (HIF1A, HDAC1, KDM1A, KDM2A) and downstream signalling markers (CA9, VEGFA, TWIST1) also increased in the EGFRHIGH subcluster (bottom). E) Volcano plot of top 50 genes in EGFRHIGH tumor subcluster analysed by log2 fold change (FC) against p-values. Red dots indicating transcripts with significantly increased fold changes including MET, PD-L1 and MET-regulated genes. F) Expression of STAT-4/PD-L1 (MET, STAT4, CD274), MET regulated (BACE2, STK40, PRSS23, DPYD, CAV1, S100A4, PYGL) and MET related immune checkpoints (HAVCR2, CD276) generally increased in the EGFRHIGH tumor subcluster (left). Expression of MET-related markers in different treatment groups (right).

## Slide 2
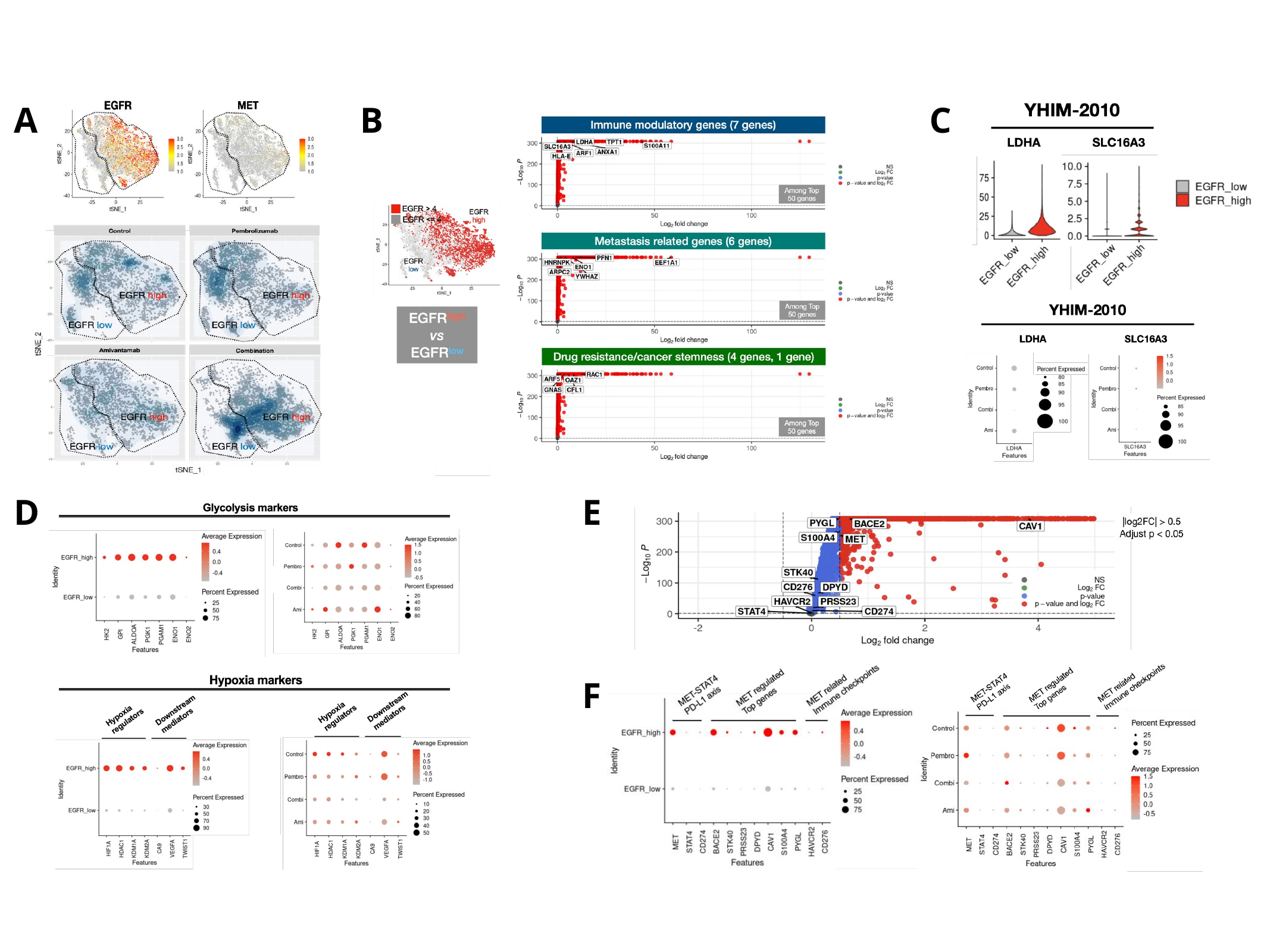

A
B
C
D
E
F
