# Supplementary material for: Exploration of Immune-Modulatory Effects of Amivantamab in Combination with Pembrolizumab in Lung and Head and Neck Squamous Cell Carcinoma
Source: Cancer Res Commun. 2024 Jul 17;4(7):1748–64. doi: 10.1158/2767-9764.CRC-24-0107 (PMC11253790; doi:10.1158/2767-9764.CRC-24-0107)
Supplement: Supplementary Figure 5 — This figure shows the selection of cell lines used for in vitro evaluation of the effects of amivantamab on the protein and surface levels EGFR, MET, LDHA and SLC16A3. [file crc-24-0107_supplementary_figure_5_supps5.pptx]

## Slide 1
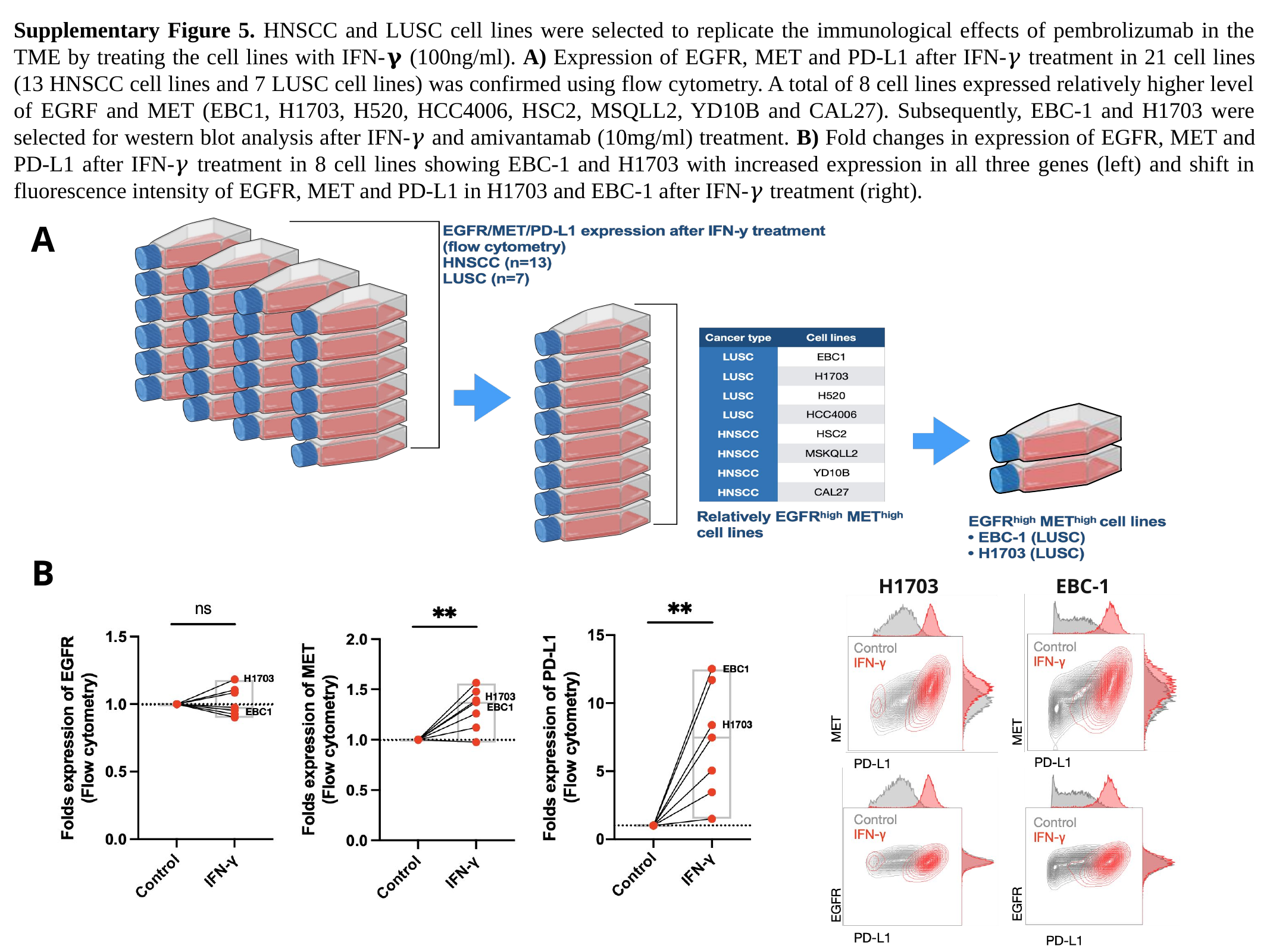

Supplementary Figure 5. HNSCC and LUSC cell lines were selected to replicate the immunological effects of pembrolizumab in the TME by treating the cell lines with IFN-𝛄 (100ng/ml). A) Expression of EGFR, MET and PD-L1 after IFN-𝛾 treatment in 21 cell lines (13 HNSCC cell lines and 7 LUSC cell lines) was confirmed using flow cytometry. A total of 8 cell lines expressed relatively higher level of EGRF and MET (EBC1, H1703, H520, HCC4006, HSC2, MSQLL2, YD10B and CAL27). Subsequently, EBC-1 and H1703 were selected for western blot analysis after IFN-𝛾 and amivantamab (10mg/ml) treatment. B) Fold changes in expression of EGFR, MET and PD-L1 after IFN-𝛾 treatment in 8 cell lines showing EBC-1 and H1703 with increased expression in all three genes (left) and shift in fluorescence intensity of EGFR, MET and PD-L1 in H1703 and EBC-1 after IFN-𝛾 treatment (right).
A
B
H1703
EBC-1
