# Supplementary material for: Exploration of Immune-Modulatory Effects of Amivantamab in Combination with Pembrolizumab in Lung and Head and Neck Squamous Cell Carcinoma
Source: Cancer Res Commun. 2024 Jul 17;4(7):1748–64. doi: 10.1158/2767-9764.CRC-24-0107 (PMC11253790; doi:10.1158/2767-9764.CRC-24-0107)
Supplement: Supplementary Table 1 — This table entails staining panels of antibodies used for flow cytometry analysis of PDX tumor samples. [file crc-24-0107_supplementary_table_1_suppst1.pptx]

## Slide 1
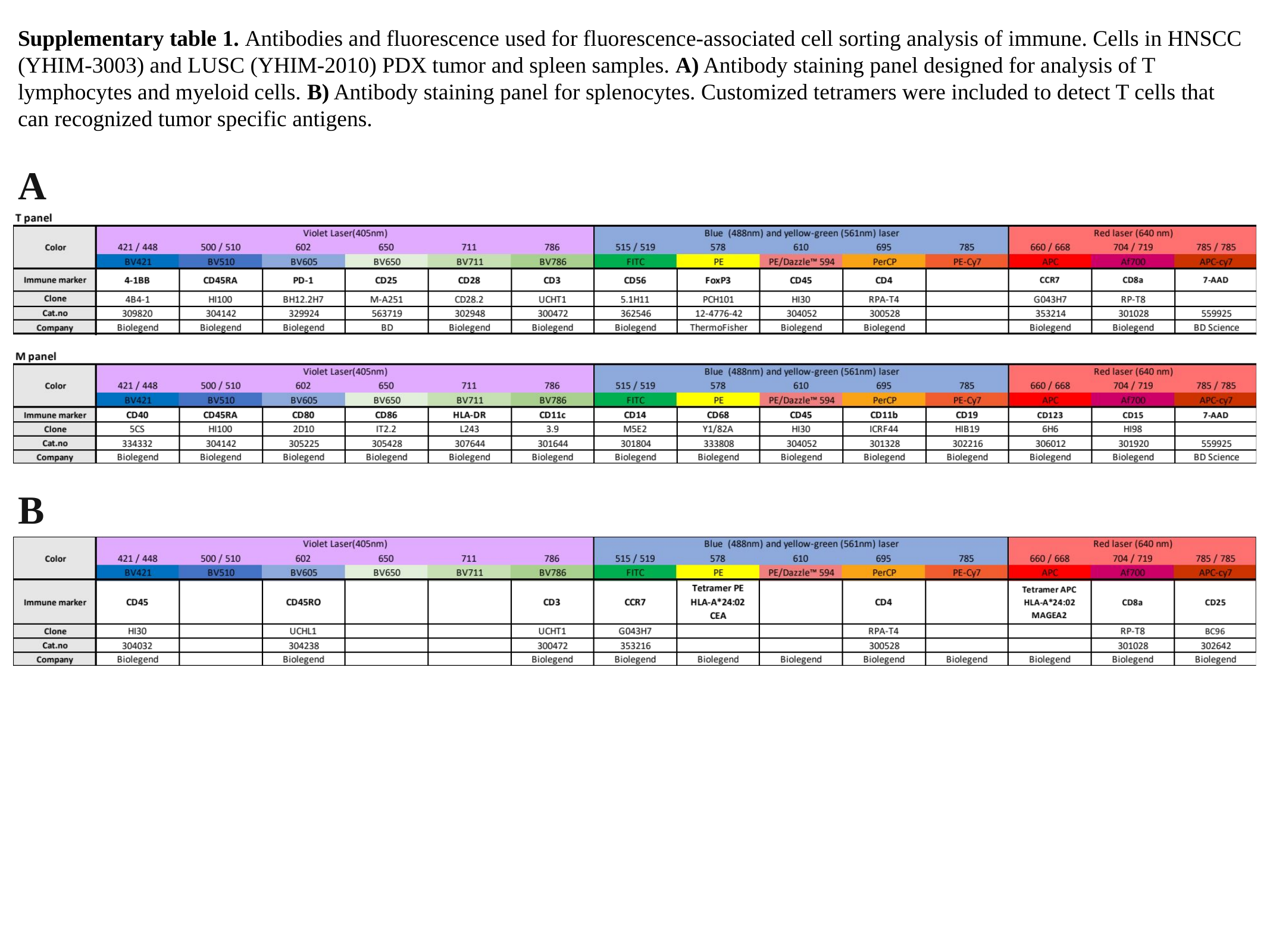

Supplementary table 1. Antibodies and fluorescence used for fluorescence-associated cell sorting analysis of immune. Cells in HNSCC (YHIM-3003) and LUSC (YHIM-2010) PDX tumor and spleen samples. A) Antibody staining panel designed for analysis of T lymphocytes and myeloid cells. B) Antibody staining panel for splenocytes. Customized tetramers were included to detect T cells that can recognized tumor specific antigens.
A
B
