# Supplementary material for: Exploration of Immune-Modulatory Effects of Amivantamab in Combination with Pembrolizumab in Lung and Head and Neck Squamous Cell Carcinoma
Source: Cancer Res Commun. 2024 Jul 17;4(7):1748–64. doi: 10.1158/2767-9764.CRC-24-0107 (PMC11253790; doi:10.1158/2767-9764.CRC-24-0107)
Supplement: Supplementary Data 1 Legend — Data legend for supplementary data 1, showing the process chart and raw whole slide images of multiplex IHC. [file crc-24-0107_supplementary_data_1_legend_suppsd1.docx]

**Supplementary Data 1 legend**

**Supplementary Data 1.** Workflow process of steps and tools used for analysis of multiplex immunohistochemistry (mIHC) whole slide images of humanized head and neck squamous cell carcinoma (HNSCC) PDX model. Whole slide images of tumor with T lymphocyte immune markers in each treatment group (n = 5) are shown. Tumor tissues were stained with antibodies, using the automated staining system (BOND Rx, Leica Biosystems) in conjunction with the Opal 7-color automation IHC kit (Akoya Biosciences), for identification of cell types and expressions of PD-1, and Granzyme B: anti-CD4 for CD4^+^ T cell (■red), anti-Pan-CK for cancer cell (■cyan blue), anti-CD8 for CD8^+^ T cell (■green), anti-GZMB for granzyme B (■yellow), anti-FoxP3 for regulatory T cell (■magenta), and anti-PD-1 for PD-1 (■orange). Approximately 33 – 98 regions of interests (ROIs) were used for analysis. Representative ROIs shown for each individual mouse.
