# Supplementary material for: Exploration of Immune-Modulatory Effects of Amivantamab in Combination with Pembrolizumab in Lung and Head and Neck Squamous Cell Carcinoma
Source: Cancer Res Commun. 2024 Jul 17;4(7):1748–64. doi: 10.1158/2767-9764.CRC-24-0107 (PMC11253790; doi:10.1158/2767-9764.CRC-24-0107)
Supplement: Supplementary Data 1 — Individual whole slide images of HNSCC PDX tumor used for TME analysis. [file crc-24-0107_supplementary_data_1_suppsd2.pptx]

## Slide 1
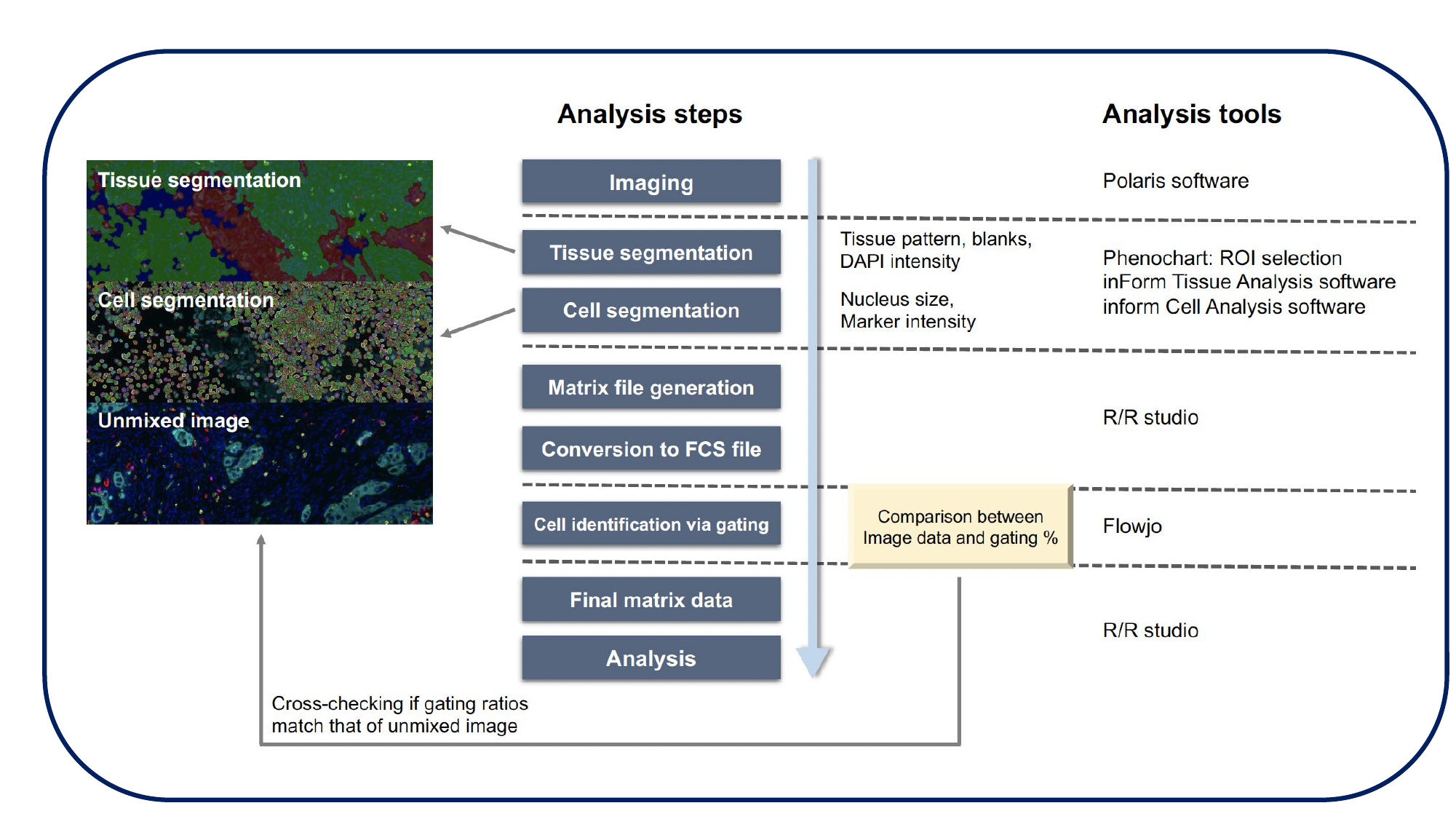

## Slide 2
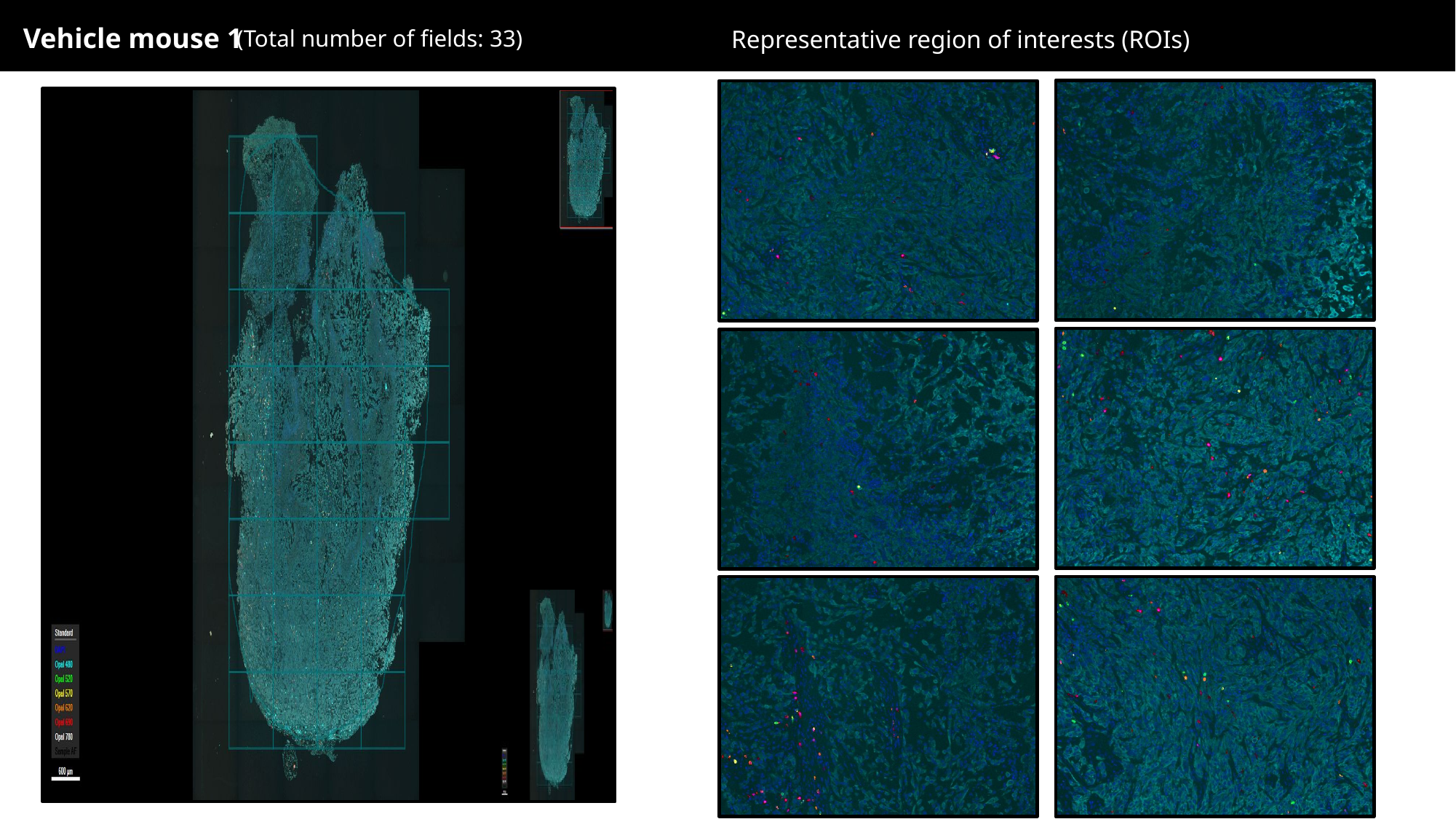

# Vehicle mouse 1
(Total number of fields: 33)
Representative region of interests (ROIs)

## Slide 3
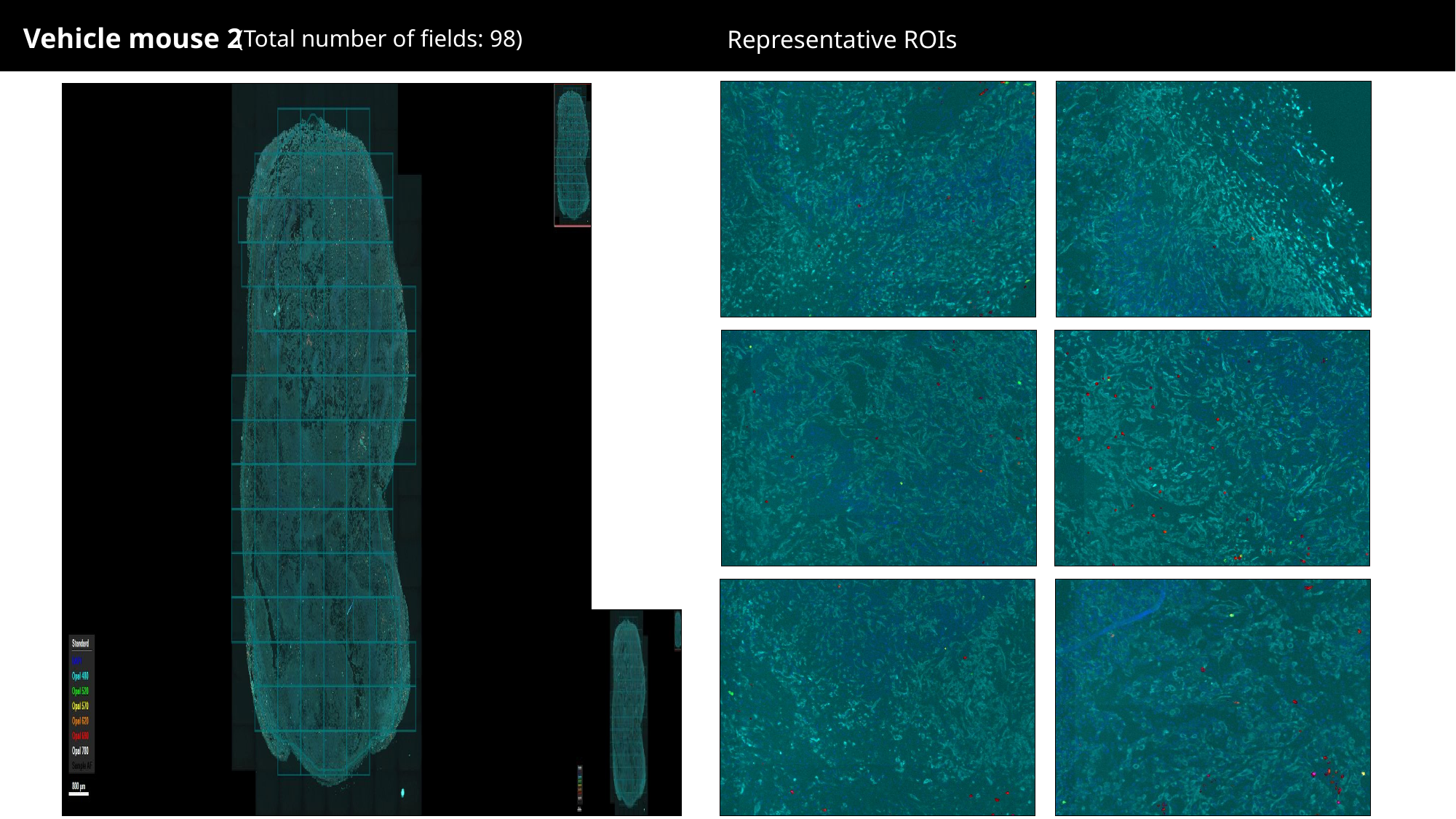

# Vehicle mouse 2
(Total number of fields: 98)
Representative ROIs

## Slide 4
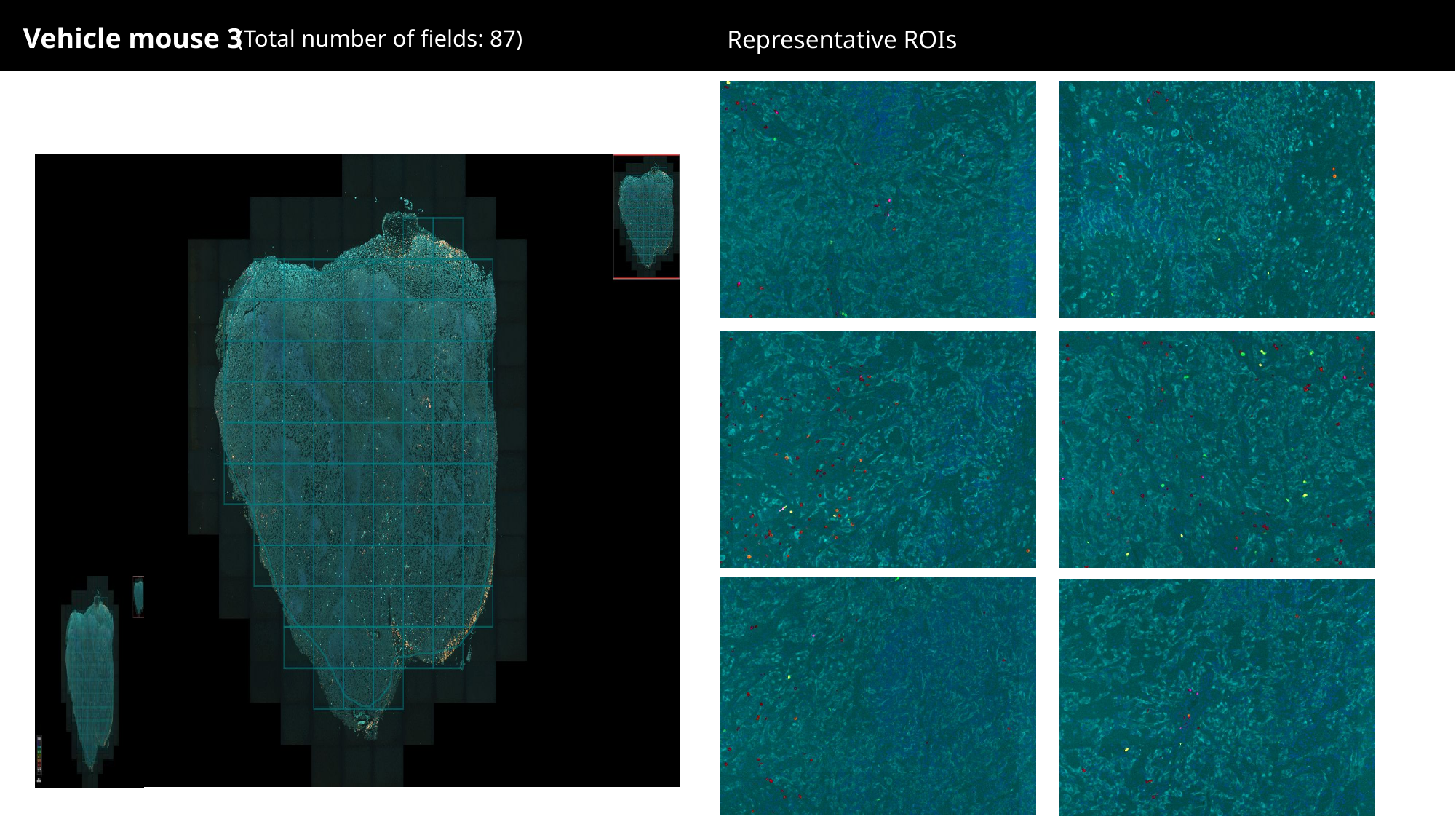

# Vehicle mouse 3
(Total number of fields: 87)
Representative ROIs

## Slide 5
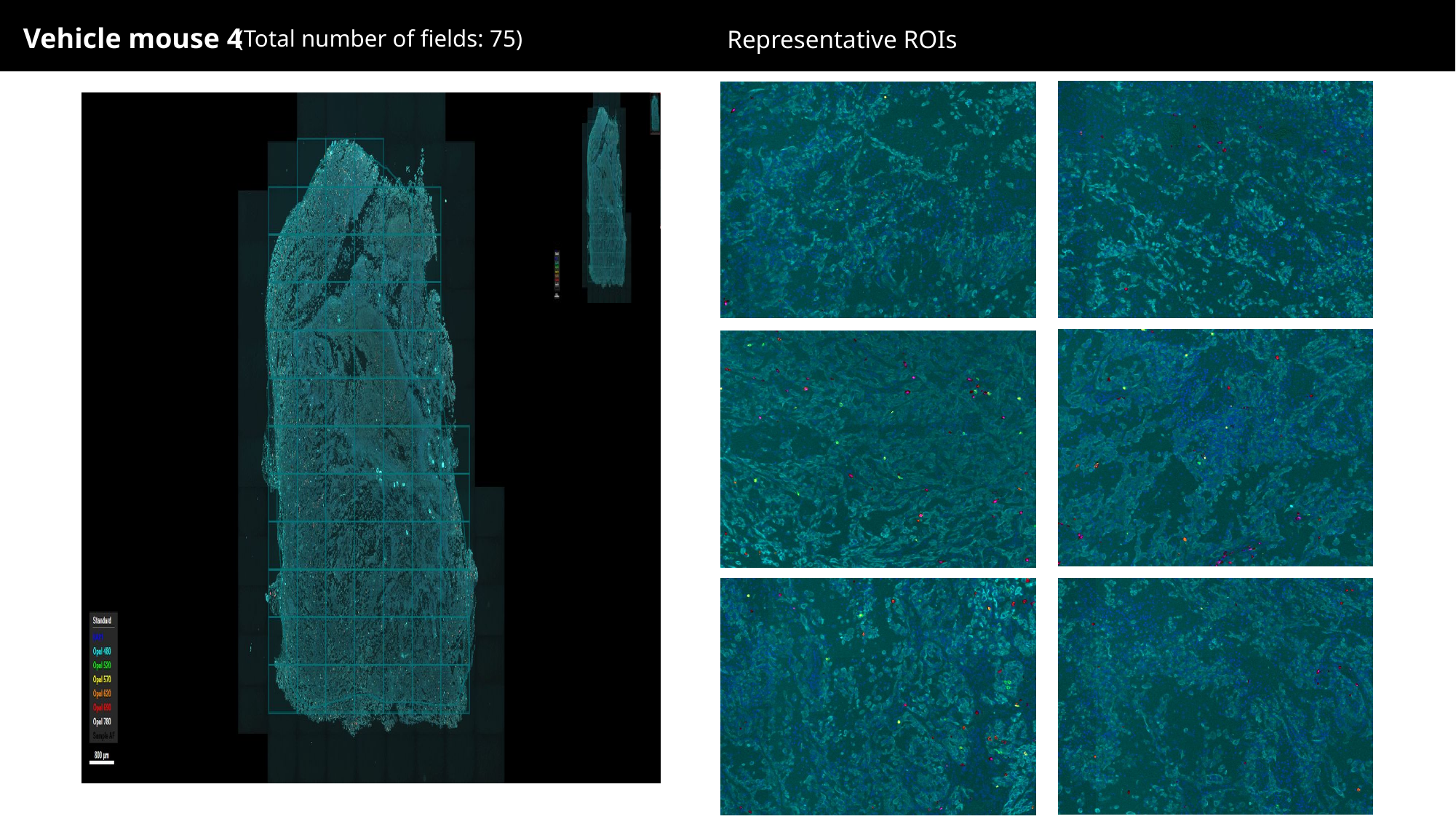

# Vehicle mouse 4
(Total number of fields: 75)
Representative ROIs

## Slide 6
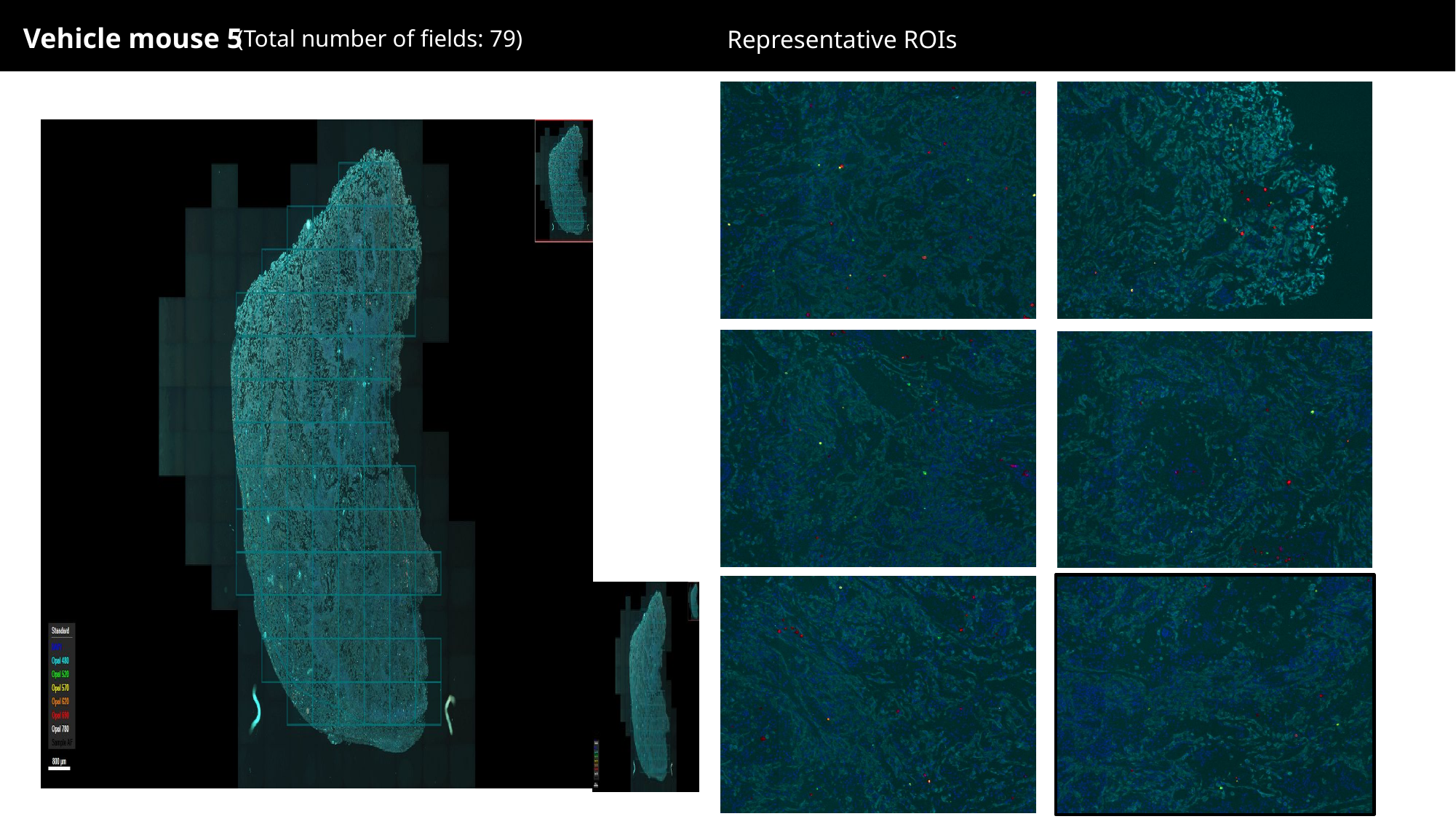

# Vehicle mouse 5
(Total number of fields: 79)
Representative ROIs

## Slide 7
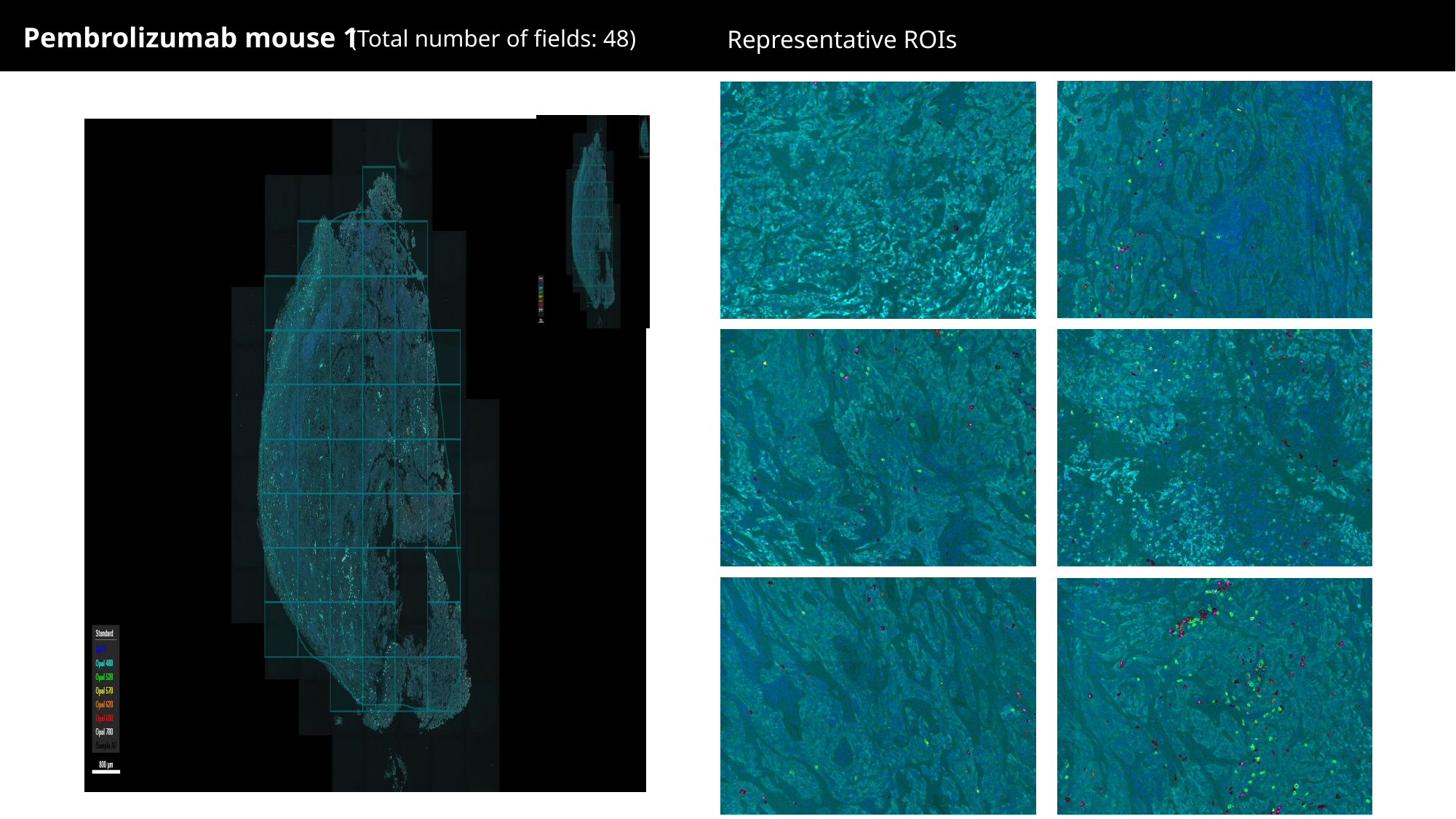

Pembrolizumab mouse 1
(Total number of fields: 48)
Representative ROIs

## Slide 8
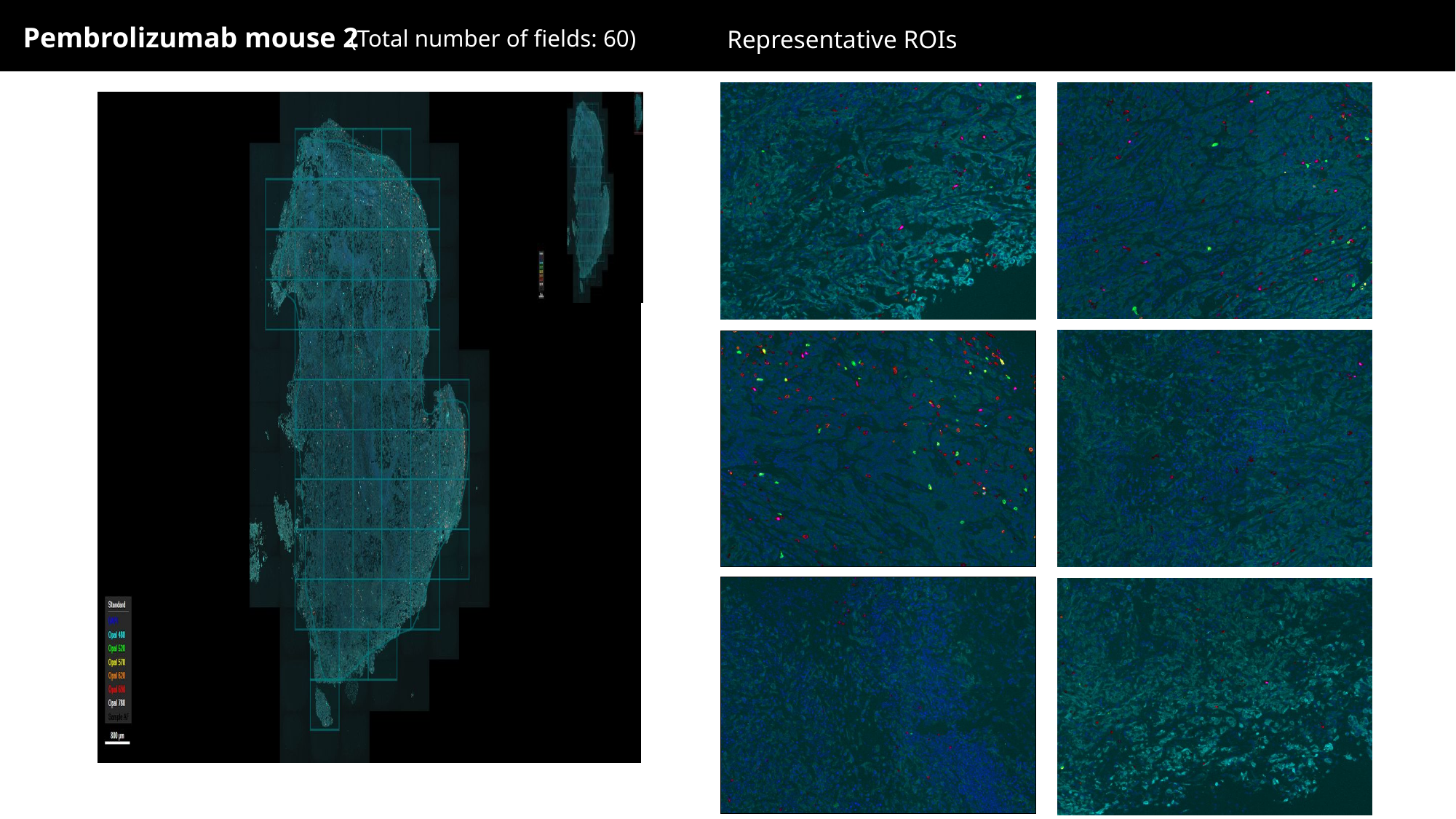

Pembrolizumab mouse 2
(Total number of fields: 60)
Representative ROIs

## Slide 9
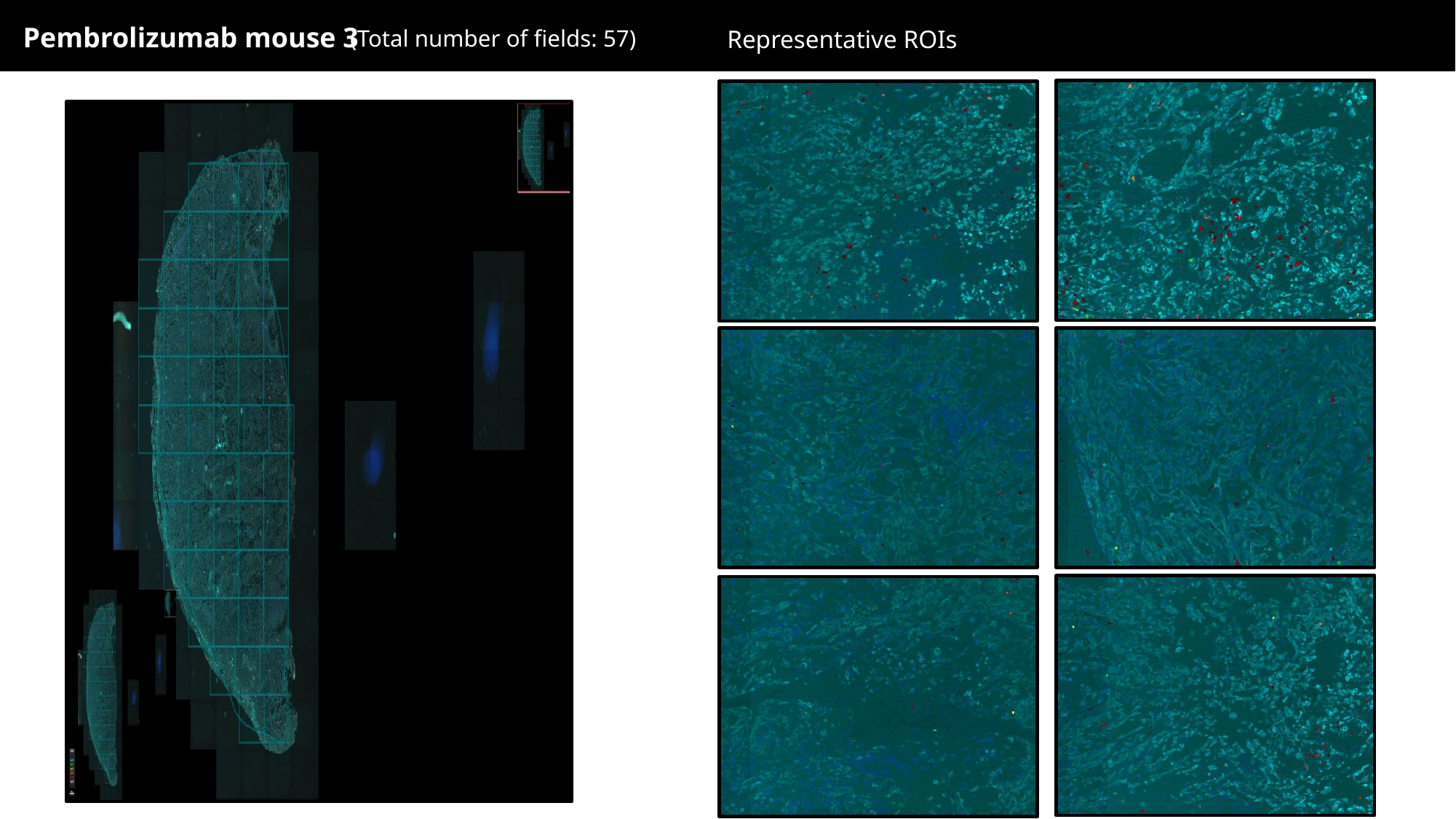

Pembrolizumab mouse 3
(Total number of fields: 57)
Representative ROIs

## Slide 10
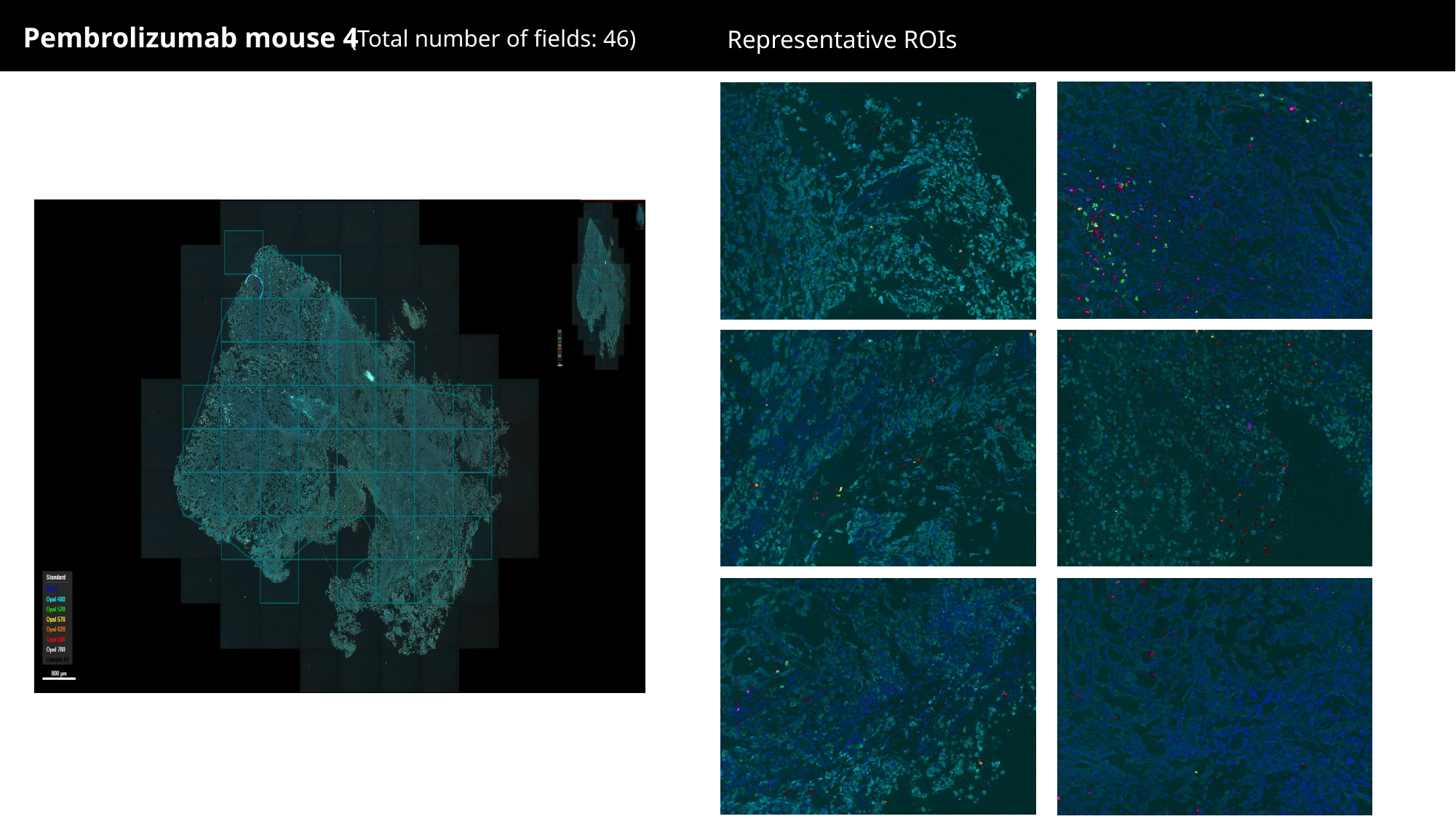

Pembrolizumab mouse 4
(Total number of fields: 46)
Representative ROIs

## Slide 11
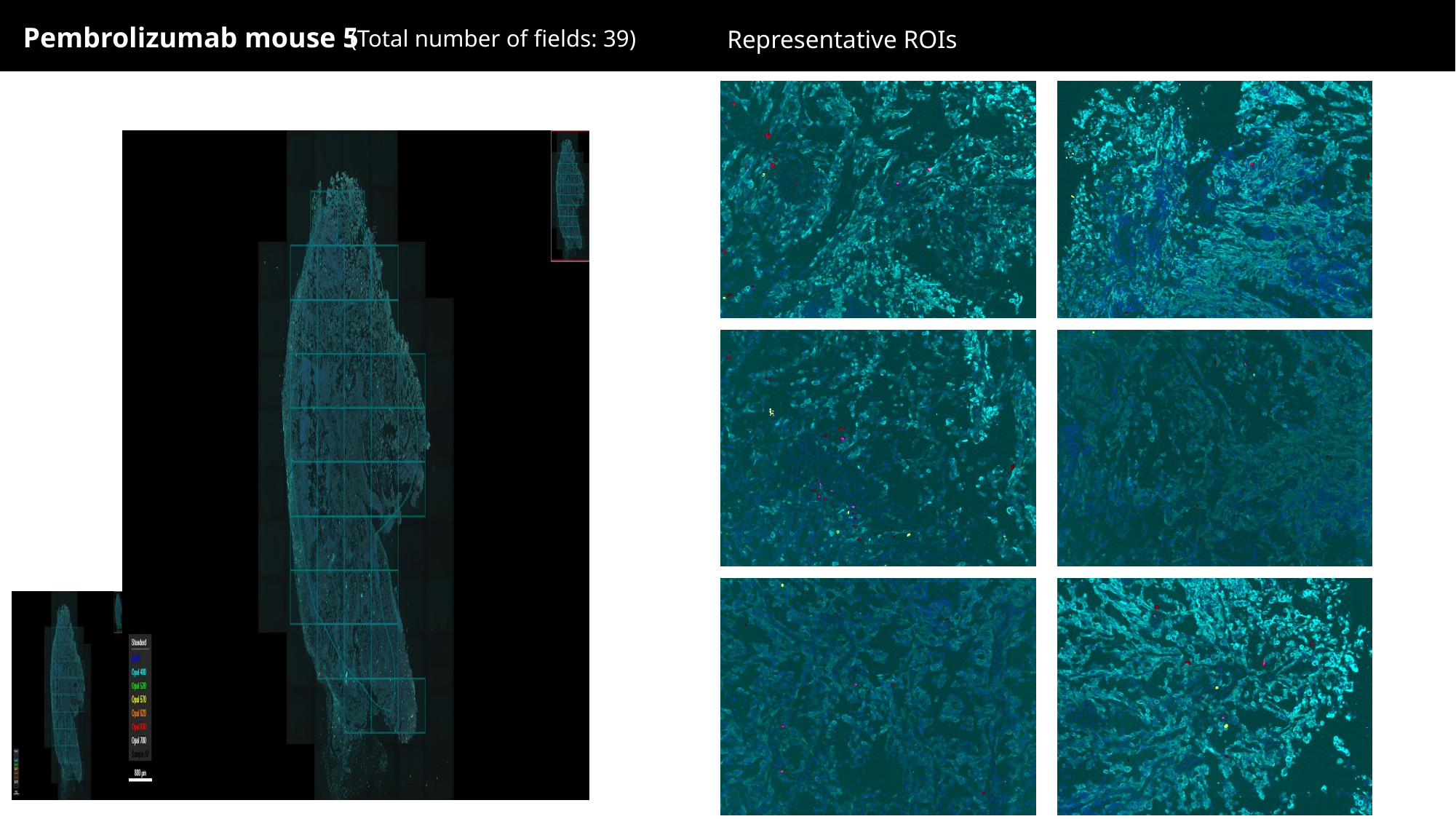

Pembrolizumab mouse 5
(Total number of fields: 39)
Representative ROIs

## Slide 12
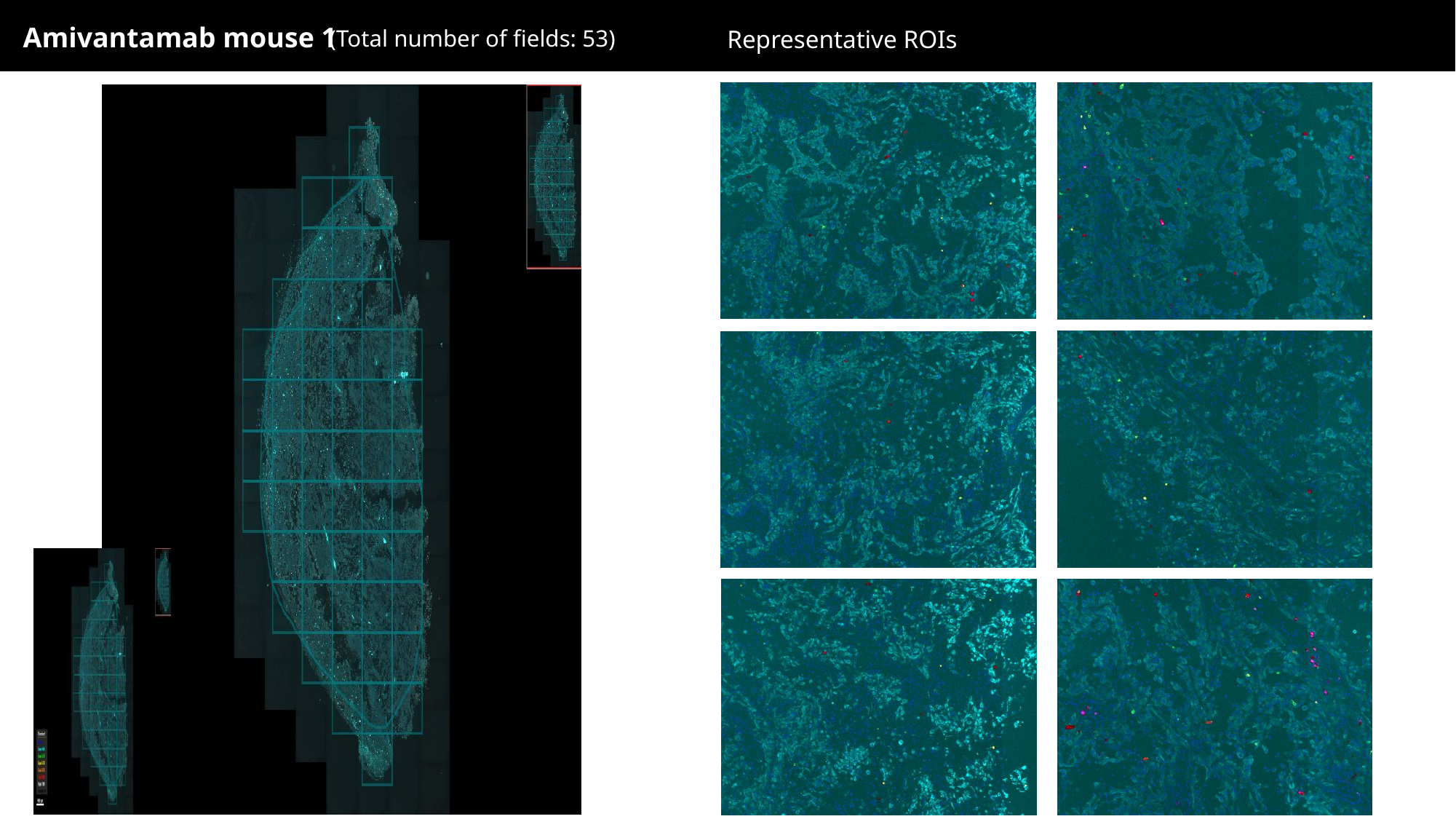

Amivantamab mouse 1
(Total number of fields: 53)
Representative ROIs

## Slide 13
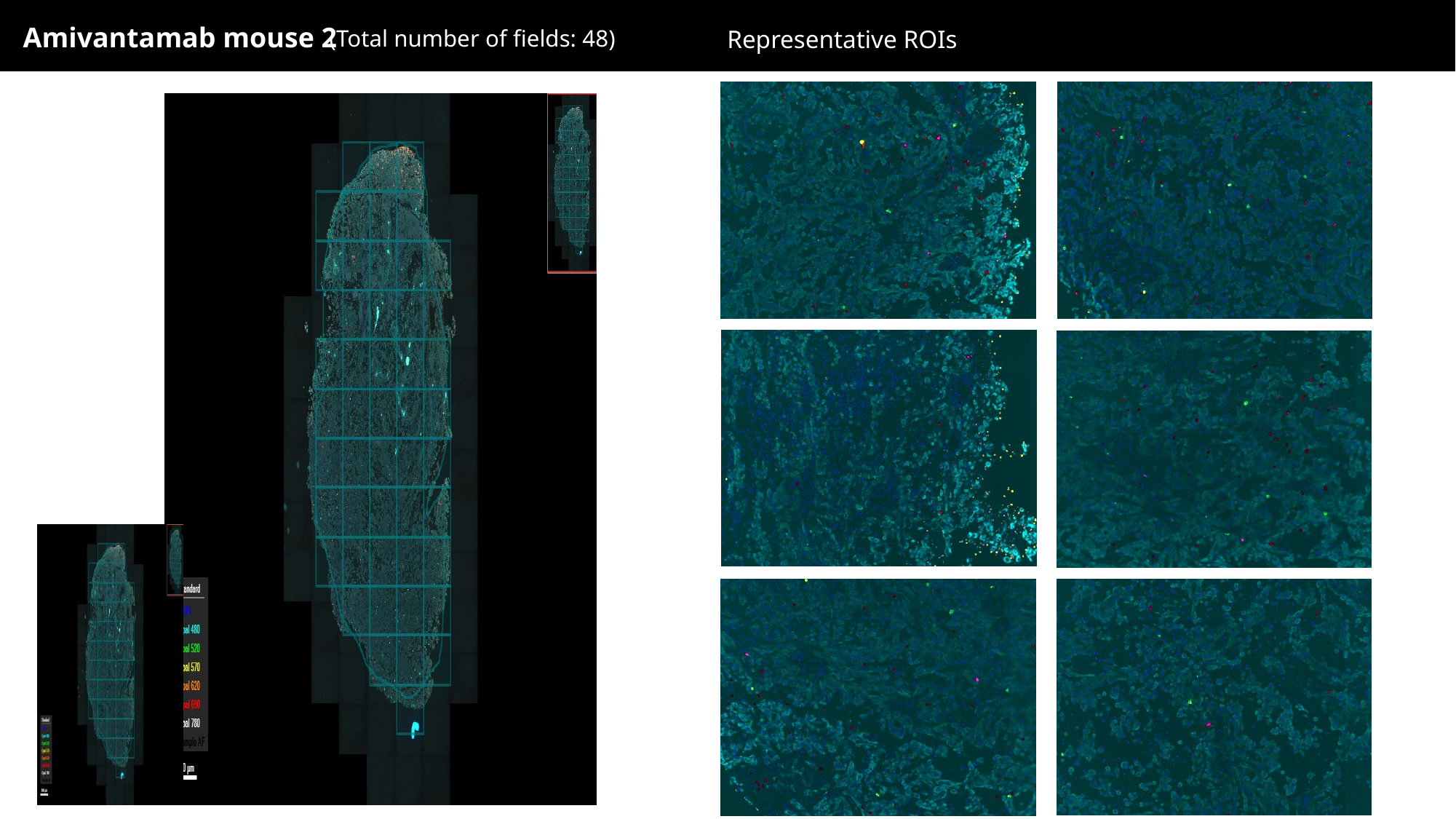

Amivantamab mouse 2
(Total number of fields: 48)
Representative ROIs

## Slide 14
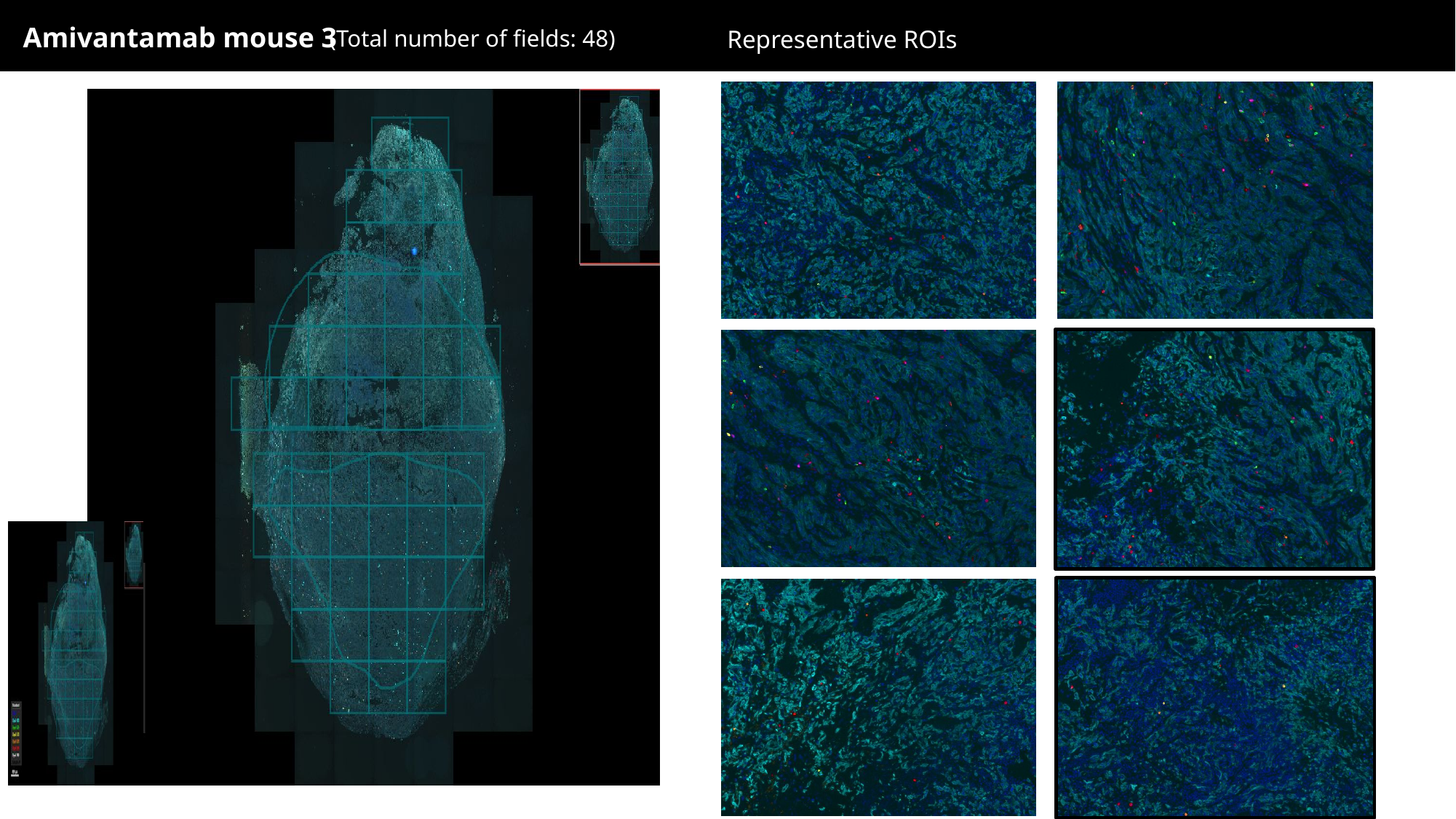

Amivantamab mouse 3
(Total number of fields: 48)
Representative ROIs

## Slide 15
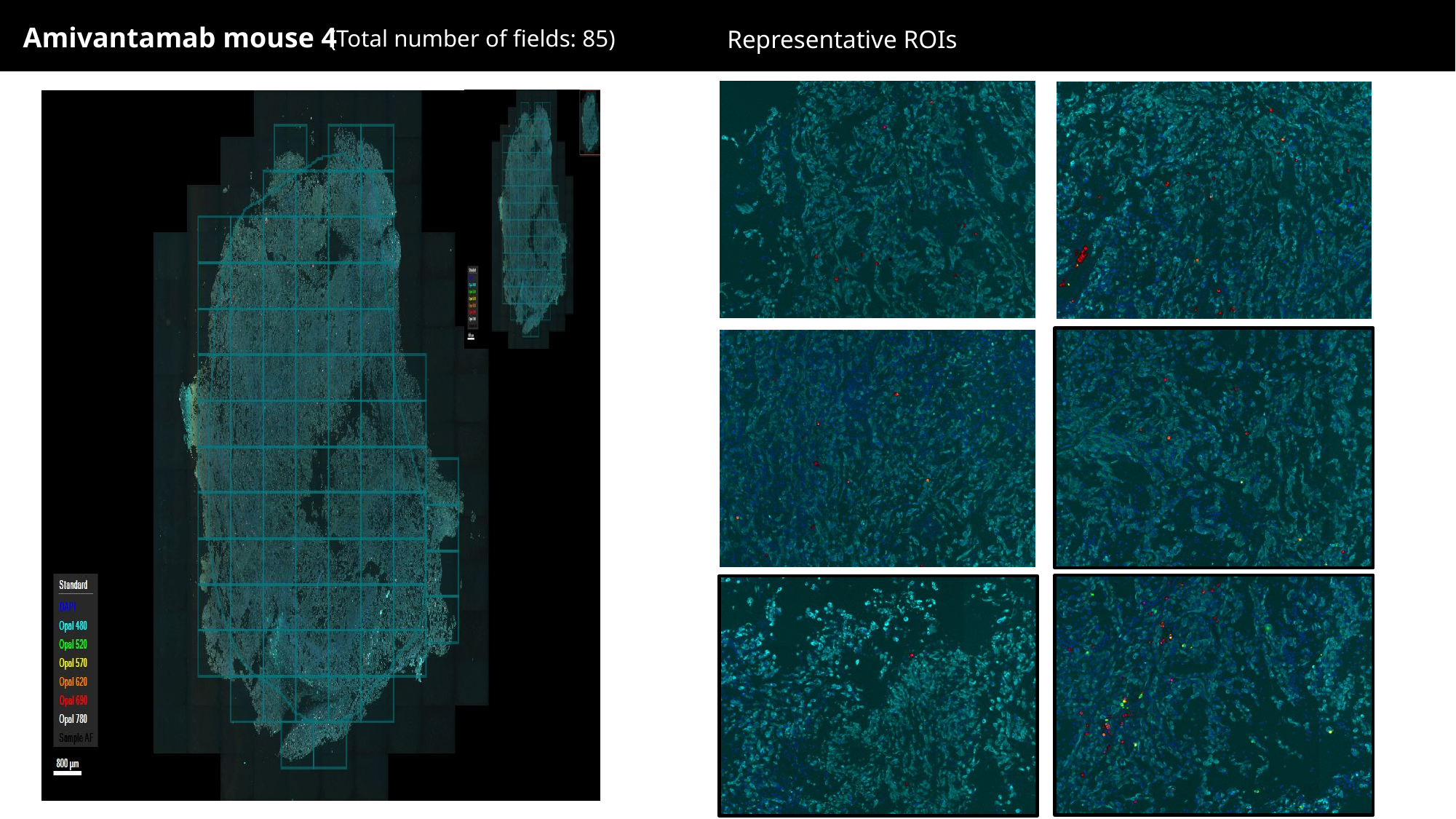

Amivantamab mouse 4
(Total number of fields: 85)
Representative ROIs

## Slide 16
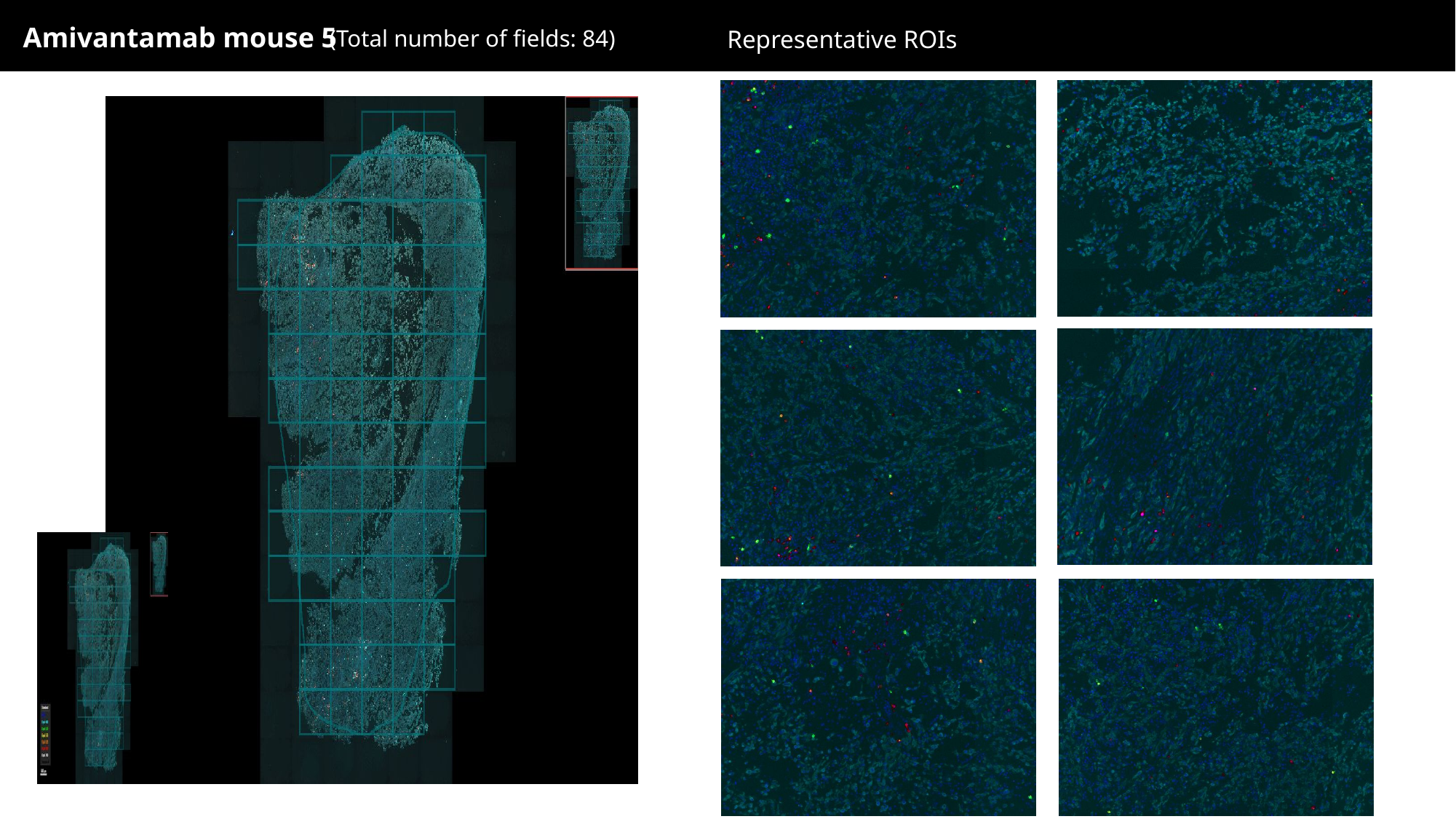

Amivantamab mouse 5
(Total number of fields: 84)
Representative ROIs

## Slide 17
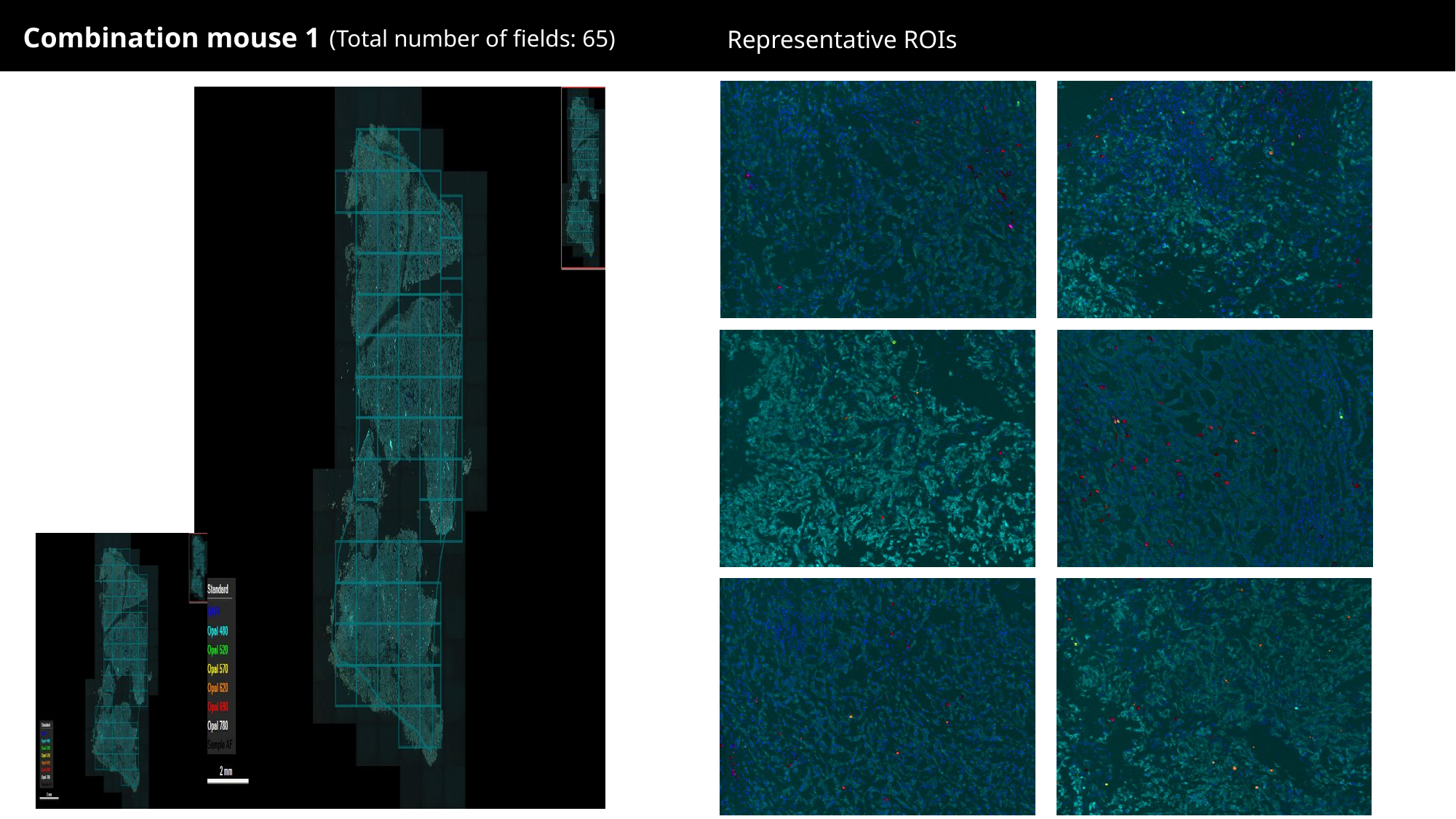

Combination mouse 1
(Total number of fields: 65)
Representative ROIs

## Slide 18
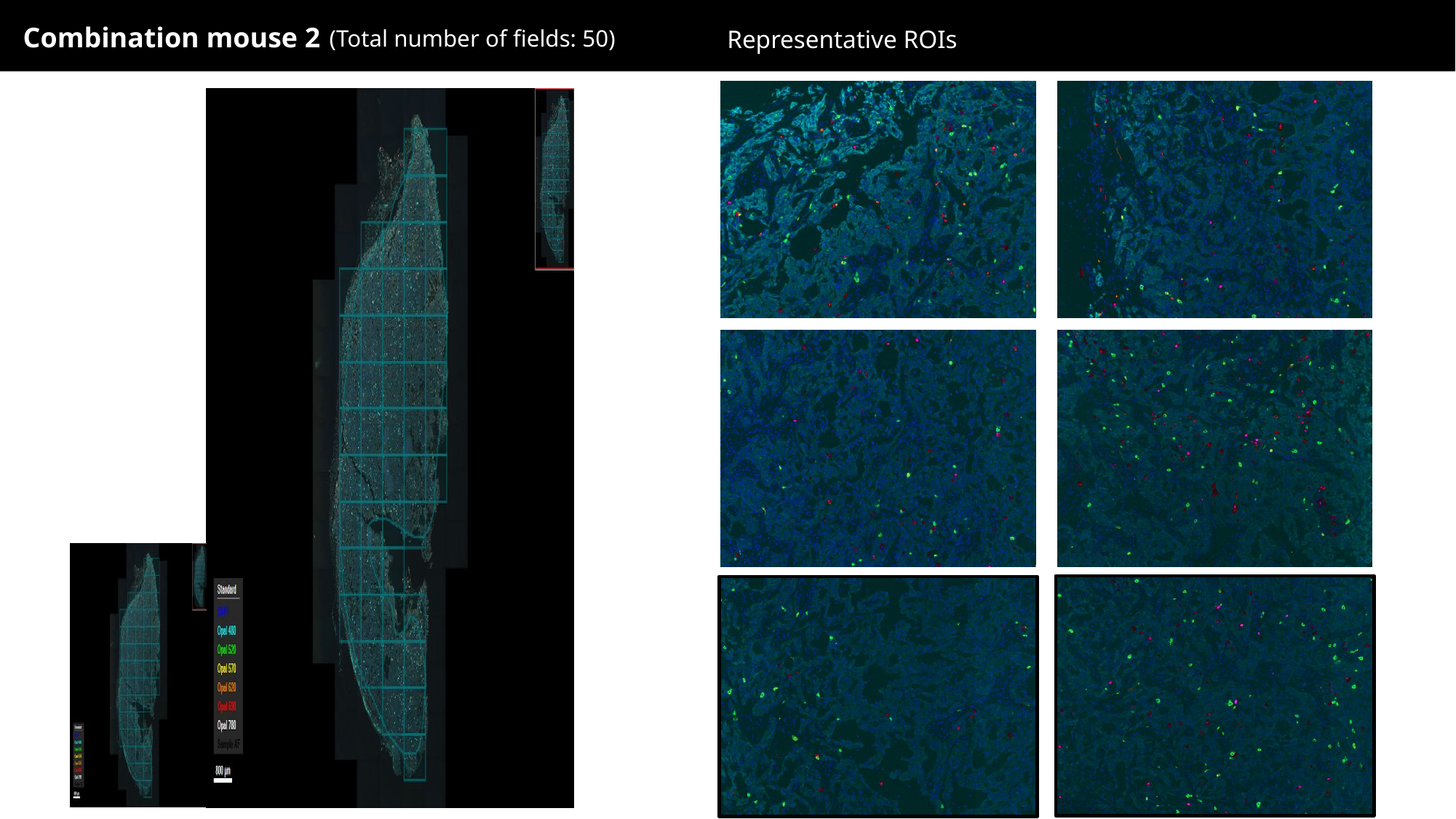

Combination mouse 2
(Total number of fields: 50)
Representative ROIs

## Slide 19
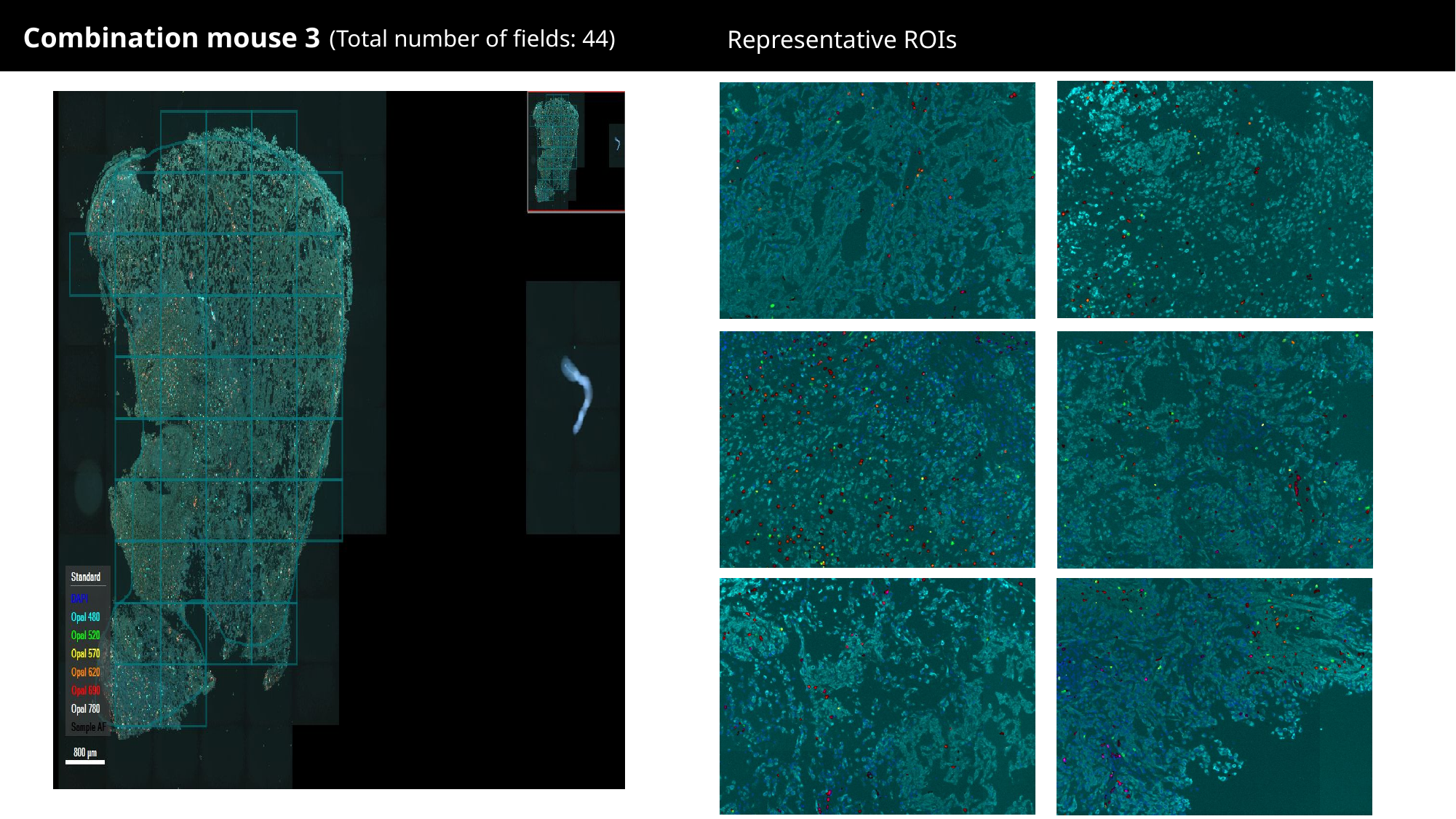

Combination mouse 3
(Total number of fields: 44)
Representative ROIs

## Slide 20
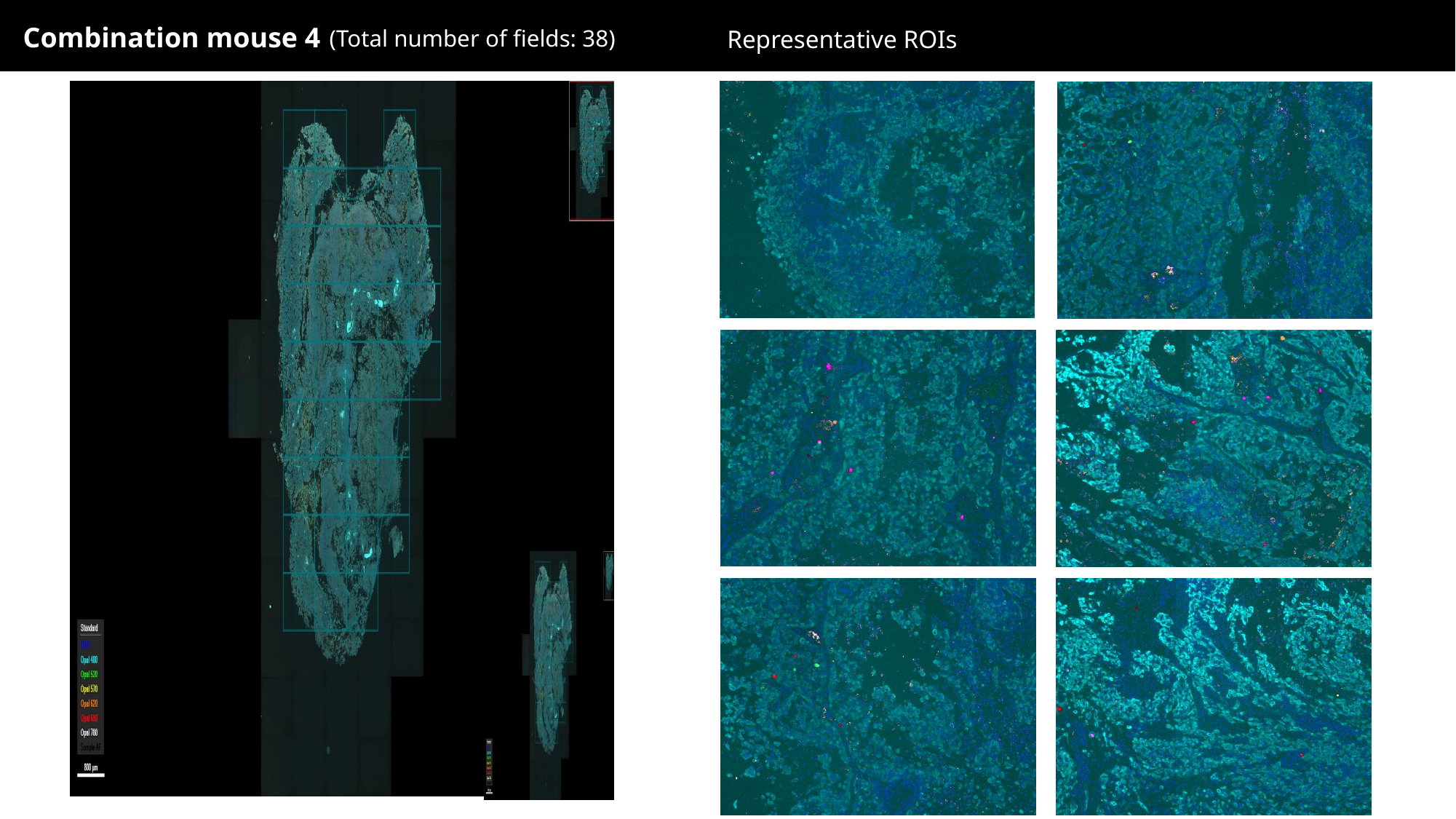

Combination mouse 4
(Total number of fields: 38)
Representative ROIs

## Slide 21
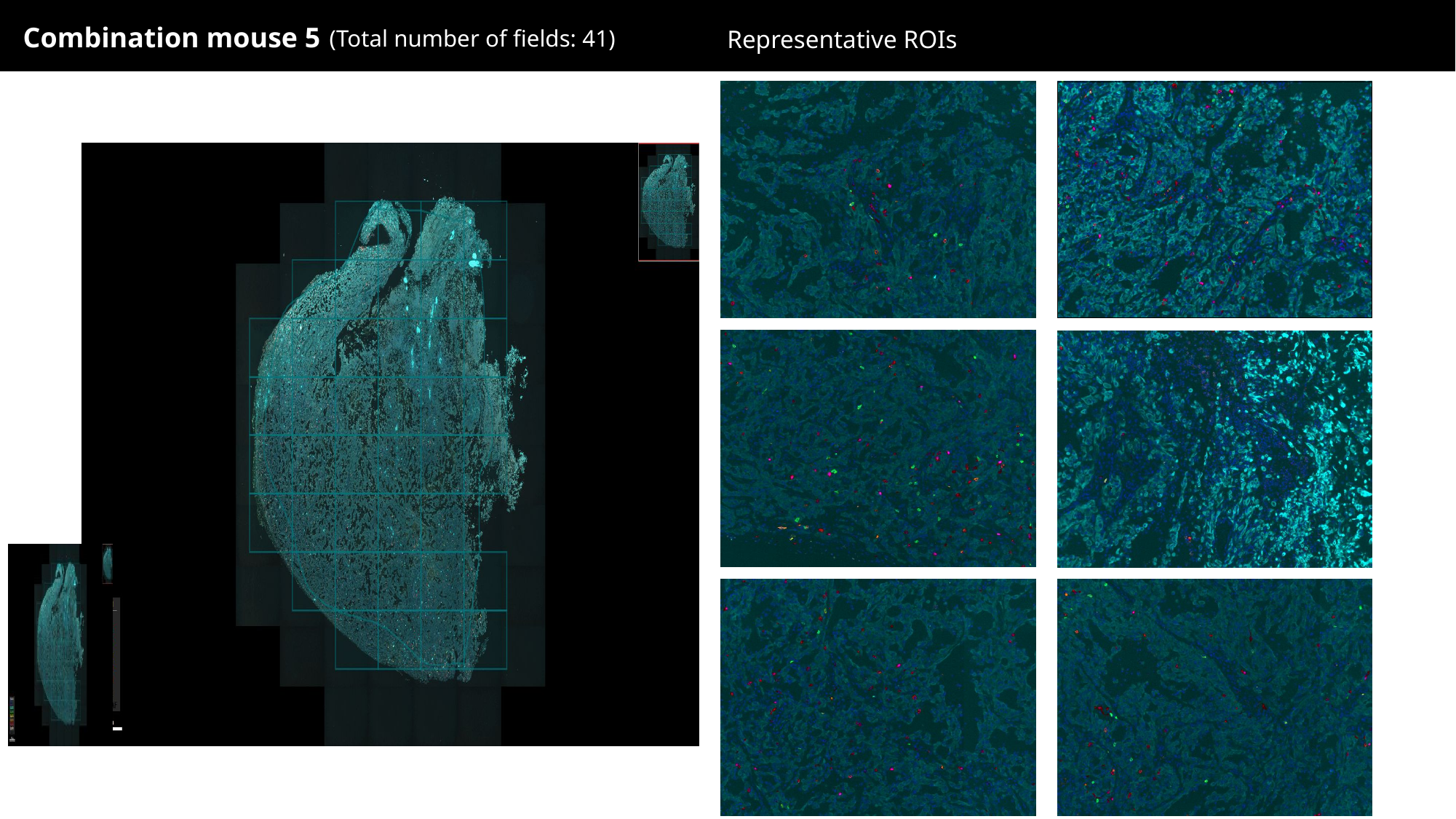

Combination mouse 5
(Total number of fields: 41)
Representative ROIs
